# Rab8a vesicles regulate Wnt ligand delivery and Paneth cell maturation at the intestinal stem cell niche

**DOI:** 10.1242/dev.121046

**Published:** 2015-06-15

**Authors:** Soumyashree Das, Shiyan Yu, Ryotaro Sakamori, Pavan Vedula, Qiang Feng, Juan Flores, Andrew Hoffman, Jiang Fu, Ewa Stypulkowski, Alexis Rodriguez, Radek Dobrowolski, Akihiro Harada, Wei Hsu, Edward M. Bonder, Michael P. Verzi, Nan Gao

**Affiliations:** 1 Department of Biological Sciences, Rutgers University, Newark, NJ 07102, USA; 2 Department of Genetics, Human Genetics Institute of New Jersey, Rutgers University, Piscataway, NJ 08854, USA; 3 Department of Biomedical Genetics, Center for Oral Biology, James P. Wilmot Cancer Center, Stem Cell and Regenerative Medicine Institute, University of Rochester Medical Center, Rochester, NY 14642, USA; 4 Department of Cell Biology, Graduate School of Medicine, Osaka University 2-2, Yamadaoka, Suita, Osaka 565-0871, Japan; 5 Rutgers Cancer Institute of New Jersey, New Brunswick, NJ 08901, USA

**Keywords:** Rab8a, Gpr177, Wntless, Wnt secretion, Intestinal stem cell, Paneth cell

## Abstract

Communication between stem and niche supporting cells maintains the homeostasis of adult tissues. Wnt signaling is a crucial regulator of the stem cell niche, but the mechanism that governs Wnt ligand delivery in this compartment has not been fully investigated. We identified that Wnt secretion is partly dependent on Rab8a-mediated anterograde transport of Gpr177 (wntless), a Wnt-specific transmembrane transporter. Gpr177 binds to Rab8a, depletion of which compromises Gpr177 traffic, thereby weakening the secretion of multiple Wnts. Analyses of generic Wnt/β-catenin targets in *Rab8a* knockout mouse intestinal crypts indicate reduced signaling activities; maturation of Paneth cells – a Wnt-dependent cell type – is severely affected. *Rab8a* knockout crypts show an expansion of Lgr5^+^ and Hopx^+^ cells *in vivo*. However, *in vitro*, the knockout enteroids exhibit significantly weakened growth that can be partly restored by exogenous Wnts or Gsk3β inhibitors. Immunogold labeling and surface protein isolation identified decreased plasma membrane localization of Gpr177 in *Rab8a* knockout Paneth cells and fibroblasts. Upon stimulation by exogenous Wnts, Rab8a-deficient cells show ligand-induced Lrp6 phosphorylation and transcriptional reporter activation. Rab8a thus controls Wnt delivery in producing cells and is crucial for Paneth cell maturation. Our data highlight the profound tissue plasticity that occurs in response to stress induced by depletion of a stem cell niche signal.

## Abstract (precis)

**Summary:** In maturing mouse Paneth cells, Wnt secretion is partly dependent on a Rab8a-mediated anterograde transport of Gpr177. Rab8a is required for Paneth cell maturation.

## INTRODUCTION

Wnts are cysteine-rich glycolipoproteins that act as paracrine or autocrine ligands believed to engage in short-range signaling (Willert et al., 2003; Willert and Nusse, 2012). Signal transduction in the Wnt-responding cell is initiated by binding of Wnts to their seven-pass transmembrane Frizzled (Fzd) receptors (Schulte, 2010; Schulte and Bryja, 2007; Wu and Nusse, 2002). The Wnt-Fzd complex recruits the cytoplasmic protein Dishevelled (Dvl, or Dsh) (Wong et al., 2003), in association with low-density lipoprotein receptor-related protein 5 and 6 (Lrp5/6), triggering the assembly of a multiprotein complex at the plasma membrane (Bilic et al., 2007). This plasma membrane-localized protein aggregate, which is sometimes referred to as the ‘Wnt signalosome’ (Bilic et al., 2007), inactivates a cytoplasmic destruction machinery consisting of casein kinase 1, glycogen synthase kinase 3 (Gsk3), axis inhibitor (Axin), adenomatosis polyposis coli and the E3 ubiquitin ligase β-Trcp (Btrc), causing β-catenin stabilization (Cadigan and Peifer, 2009; Huang and He, 2008; MacDonald et al., 2009) and transcriptional activation of Wnt targets (He et al., 2004; MacDonald and He, 2012; Tamai et al., 2000; Wehrli et al., 2000). This signaling cascade, which is often referred to as the canonical Wnt pathway, plays a fundamental role in fetal development and adult tissue homeostasis (Clevers, 2006; Clevers and Nusse, 2012). Inappropriate activation of this pathway in diseases, especially colon cancers, has highlighted its profound influence on cellular behavior (Angers and Moon, 2009; Clevers and Nusse, 2012; de Lau et al., 2007; MacDonald et al., 2009; Nusse et al., 2008; Polakis, 2007; Reya and Clevers, 2005). Certain Wnt-Fzd complexes activate non-canonical Wnt pathways and regulate cell migration and polarity via Rho subfamily small GTPases (Boutros and Mlodzik, 1999; Eaton et al., 1996; Fanto et al., 2000; Habas et al., 2003, 2001; Sakamori et al., 2014; Strutt et al., 1997; Wallingford and Habas, 2005).

In Wnt-producing cells, newly synthesized Wnt proteins are lipid modified in the endoplasmic reticulum (ER) by an acyltransferase, Porcupine (Takada et al., 2006), and transported by the multi-pass transmembrane protein Wntless [also known as G protein-coupled receptor 177 (Gpr177) in mammals] for exocytosis (Bänziger et al., 2006; Bartscherer et al., 2006). Global or tissue-specific ablation of Wntless/Gpr177 in various animals causes phenotypes that resemble loss of Wnts (Bänziger et al., 2006; Bartscherer et al., 2006; Fu et al., 2011), leading to the current notion that Wntless/Gpr177 represents the specific and possibly sole transporter for secretion of most Wnts (Ching and Nusse, 2006; Das et al., 2012; Port and Basler, 2010). After Wnt release at the cell surface, Gpr177 is internalized from the plasma membrane via a Clathrin-dependent pathway to endosomes (Gasnereau et al., 2011; Pan et al., 2008), where it is retrieved in a retromer Vps35- and Snx3-dependent fashion to the Golgi for new rounds of Wnt transport (Belenkaya et al., 2008; Franch-Marro et al., 2008; Harterink et al., 2011; Port et al., 2008; Rojas et al., 2008; Yang et al., 2008). In contrast to the intensive study of retrograde traffic of Gpr177, the cellular machineries that govern anterograde Gpr177-Wnt transport have not been fully explored. It is unclear whether Gpr177-Wnt follows a vesicular membrane bulk flow (Hong and Tang, 1993) or if Wnt exocytosis is subject to regulation by specific secretory machinery (Das et al., 2012).

Among various cell types that produce distinct cohorts of Wnts surrounding the intestinal stem cell niche (Gregorieff et al., 2005), Paneth cells are the major epithelial Wnt producers, expressing Wnt3, Wnt6 and Wnt9b, and co-occupy the crypt bottom with stem cells. The self-renewal of fast-cycling Lgr5^+^ stem cells (Barker et al., 2007), as shown by organoid-forming capacity in culture, is enhanced by close association with Paneth cells or by addition of exogenous Wnt ligands (Sato et al., 2011). The ablation of Paneth cells in several mouse models caused recoverable loss of Lgr5^+^ stem cells (Sato et al., 2011). However, Atoh1-deficient mouse intestines with an absence of Paneth cell differentiation preserved functional intestinal epithelia (Durand et al., 2012; Kim et al., 2012), hinting at a high plasticity of crypt cells (Tetteh et al., 2014). In addition to Paneth cells, subepithelial stromal cells express Wnt2b, Wnt4 and Wnt5a (Farin et al., 2012; Gregorieff et al., 2005; Miyoshi et al., 2012). Wnt5a^+^ mesothelial cells contribute to regenerating nascent crypts after tissue injury (Miyoshi et al., 2012). Intestinal epithelia-specific ablation of Wnt3 (Farin et al., 2012), or Porcupine deletion in both epithelia and myofibroblasts (Kabiri et al., 2014; San Roman et al., 2014), did not cause detectable tissue damage. In culture, Wnt3-deficient intestinal organoids fail to propagate, but administration of Wnts was able to restore the growth (Farin et al., 2012), collectively suggesting that multiple sources of Wnts redundantly support the stem cell niche.

Upon quantitative loss of Lgr5^+^ stem cells, a proposed ‘reserve’ stem cell pool rejuvenates the epithelia (Tian et al., 2011). These stem cells are considered to be slow-cycling and are identified by several markers, including Bmi1 (Sangiorgi and Capecchi, 2008), Hopx (Takeda et al., 2011), Lrig1 (Powell et al., 2012) and Tert (Montgomery et al., 2011). Lineage conversion from these proposed quiescent cells to Lgr5^+^ cells has been observed during homeostasis or epithelial injury (Takeda et al., 2011; Tian et al., 2011; Yan et al., 2012). However, all these markers are highly expressed in Lgr5^+^ cells (Muñoz et al., 2012; Wong et al., 2012) and also in a subset of Lgr5^+^ label-retaining cells (LRCs) (Buczacki et al., 2013). This subset of Lgr5^+^ cells was recently proposed to constitute secretory precursors for Paneth and enteroendocrine cells and could be reactivated by injury for epithelial regeneration (Buczacki et al., 2013; Roth et al., 2012). In a parallel study, Dll1^+^ secretory precursors were shown to revert to stem cells upon injury to regenerate the epithelia (van Es et al., 2012). These secretory precursors are postulated to represent the proposed ‘+4' quiescent cells (Tetteh et al., 2014). However, in contrast to Lgr5^+^ cells, Bmi1^+^ cells were shown to resist Wnt perturbation and radiation injury (Yan et al., 2012). Thus, whether a dedicated quiescent stem cell population truly exists is still under debate (Tetteh et al., 2014).

Vesicular trafficking influences Wnt signaling capacities in both ligand-producing and ligand-receiving cells (de Groot et al., 2013; Feng and Gao, 2014; Kabiri et al., 2014; San Roman et al., 2014). During intestinal differentiation, the intestinal cell fate activator Cdx2 transcriptionally regulates the expression of Rab8 small GTPases, which are members of the Ras superfamily (Gao and Kaestner, 2010). Rab8 directly binds isoforms of the myosin V motor (Hume et al., 2001; Rodriguez and Cheney, 2002; Roland et al., 2009), facilitating exocytotic cargo movements on actin tracks in epithelial and non-epithelial cells (Ang et al., 2003; Bryant et al., 2010; Gerges et al., 2004; Hattula et al., 2006; Henry and Sheff, 2008; Huber et al., 1993a,b; Sato et al., 2009; Sun et al., 2014). Global *Rab8a* ablation in mice impairs the apical delivery of peptidases and nutrient transporters to enterocyte brush borders; as a consequence, these proteins are transported into lysosomes, causing nutrient deprivation and postnatal death of knockout mice (Sato et al., 2007). However, the contribution of Rab8 vesicles to intestinal crypt homeostasis is not defined.

A recent screening for Rab modulators of the Wnt pathway identified RAB8B, but not RAB8A, as a crucial regulator of canonical Wnt signaling in receiving cells by directly interacting with LRP6 and CK1γ (Demir et al., 2013). We provide evidence here that, in Wnt-producing cells, Rab8a regulates Gpr177 anterograde traffic and Wnt secretion. Using immunogold labeling of endogenous Gpr177 in native Wnt producers, Wnt secretion and reporter assays, we demonstrate that *Rab8a* ablation impairs Gpr177 trafficking in Wnt producers, attenuating Wnt secretion and canonical Wnt signaling *in* *vivo* and *ex vivo*. *Rab8a* knockout intestinal crypts showed altered cell organization in response to decreased extracellular Wnts in the niche. These data shed light on intestinal crypt plasticity in response to stress induced by defective niche signal traffic.

## RESULTS

### Gpr177 traffics through Rab8a vesicles

**Fig. 1.**
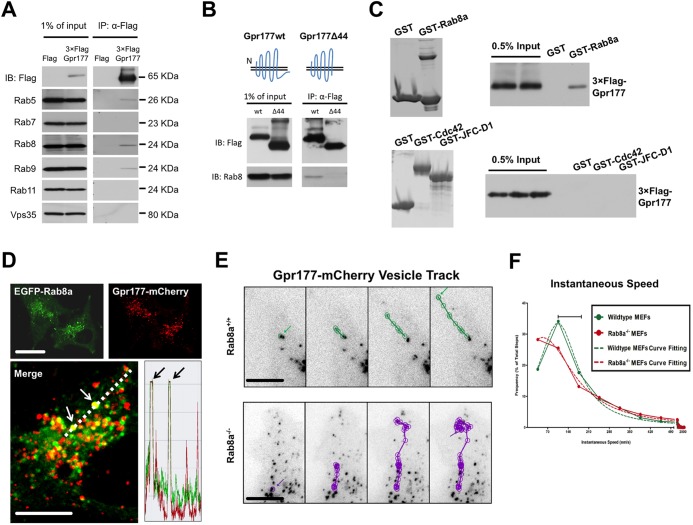
**RAB8A intersects GPR177 traffic.** (A) Flag-GPR177 was immunoprecipitated (IP) from lysates of a stable human HeLa cell line in the presence of 1% Triton X-100. Precipitates were blotted (IB) for various vesicular markers. (B) Flag-GPR177Δ44 lacking the C-terminal tail failed to co-immunoprecipitate with RAB8A. (C) GST pull-down showed binding of Flag-GPR177 to GST-RAB8A, but not to GST, GST-CDC42 or GST-JFC-D1. Data are representative of three independent experiments. (D) Live cell imaging detected GPR177-mCherry in EGFP-RAB8A vesicles in HeLa cells. The line scan histogram (along the dotted line in the merge) shows colocalization of the two fluorescent signals (arrows). (E) Single vesicle tracks of GPR177-mCherry vesicles in *Rab8a^+/+^* (top) and *Rab8a^−/−^* (bottom) MEFs (*t*=10 s; *n*=8 vesicles in each cell). Arrows indicate the start and end of an individual vesicle track. See supplementary material Fig. S1B, Movies 1 and 2. (F) Instantaneous vesicle speed distribution (nm/s), with a bin size of 70 nm/s. The speed of Gpr177 vesicles in *Rab8a^+/+^* cells peaks at 130-140 nm/s (green dashed line), agreeing with myosin V-powered movement (bar), which was reduced in *Rab8a^−/−^* MEFs (red dashed curve). 1717 steps for wild type and 995 steps for *Rab8a^−/−^* were analyzed. Scale bars: 10 μm.

We established a stable Henrietta Lacks (HeLa) human cell line expressing 3×Flag-GPR177 to identify regulators for Wnt-GPR177 trafficking. Using cell lysates extracted in the presence of 1% Triton X-100, we performed co-immunoprecipitation analyses to identify potential interactions between GPR177 and key trafficking regulators. We detected association of GPR177 with RAB5, RAB8A and RAB9 (Fig. 1A). As GPR177 is internalized into endosomes (Belenkaya et al., 2008) during retrograde trafficking, association of GPR177 with RAB5 and RAB9 reflected endocytosis of GPR177 (Gasnereau et al., 2011). Association between GPR177 and the RAB8A vesicular compartment has not been described. Given that RAB8 transports several G protein-coupled receptors (GPCRs) (Dong et al., 2010; Esseltine et al., 2012), we postulated that RAB8A vesicles might be involved in anterograde traffic of the Wnt-GPR177 complex. Of note, under similar conditions, 3×Flag-GPR177 was not detected in association with RAB7, RAB11 or VPS35 (Fig. 1A), suggesting that GPR177 and RAB8A might exist in a relatively stable detergent-resistant complex. The interaction between GPR177 and RAB8A was likely to be physiologically relevant as a truncated GPR177 lacking the C-terminal cytoplasmic tail (GPR177Δ44) failed to associate with RAB8A (Fig. 1B). Using glutathione S-transferase (GST)-RAB8A fusion proteins, we performed GST pull-down assays using 3×Flag-GPR177 cell lysates and consistently detected binding of GPR177 to GST-RAB8A but not to GST, GST-CDC42 or GST-synaptotagmin-like 1 (JFC)-D1 (Fig. 1C), suggesting that RAB8A and GPR177 indeed associate in a complex. When GPR177-mCherry and EGFP-RAB8A were transiently expressed in HeLa cells (Fig. 1D) or human colonic epithelial Caco2 cells (supplementary material Fig. S1A), three populations of vesicles – mCherry positive, EGFP positive and mCherry/EGFP double positive – were observed and confirmed by line scans, indicating that some GPR177 traffics through RAB8A vesicles (Fig. 1D).

Rab8a is required for docking vesicular cargo on the myosin V motor for exocytotic transport (Khandelwal et al., 2013; Roland et al., 2011). We derived *Rab8a^−/−^* and wild-type mouse embryonic fibroblasts (MEFs) and transiently expressed GPR177-mCherry in these cells to track dynamic vesicle movement. *Rab8a^−/−^* MEFs showed no qualitative difference in terms of peri-nuclear localization of Gpr177^+^ vesicles when compared with wild-type MEFs of similar cell morphology (Fig. 1E; supplementary material Fig. S1B). However, by binning instantaneous speeds of Gpr177 vesicles in 70 nm/s increments and analyzing the frequency distribution as a percentage of total steps (*n*=1717 for wild type and *n*=995 for *Rab8a^−/−^*; Fig. 1F; supplementary material Movies 1 and 2), we observed a reduction in the frequency of instantaneous speeds within 106-176 nm/s (centered at 140 nm/s) in *Rab8a^−/−^* MEFs. Myosin V-powered melanosome transport has been reported to have an average instantaneous speed of 140 nm/s (Wu et al., 1998). Curve fitting that identified peaks of frequency distributions showed that the instantaneous speed of Gpr177^+^ vesicles in *Rab8a^−/−^* cells decreased to 53 nm/s, as compared with 105 nm/s in *Rab8a^+/+^* cells (dashed lines, Fig. 1F). An increased number of Gpr177^+^ vesicles in the slow (1-50 nm/s) and fast (250-500 nm/s) speed ranges in *Rab8a^−/−^* cells suggested diffusion-like movement and microtubule-dependent transport, respectively (Wu et al., 1998). These results suggest that the absence of Rab8a vesicles affected Gpr177 trafficking dynamics, which was possibly attributable to the reduced myosin V-powered vesicle movement (Roland et al., 2011; Sun et al., 2014).

### Impaired Wnt secretion in *Rab8a*^−/−^ cells

**Fig. 2.**
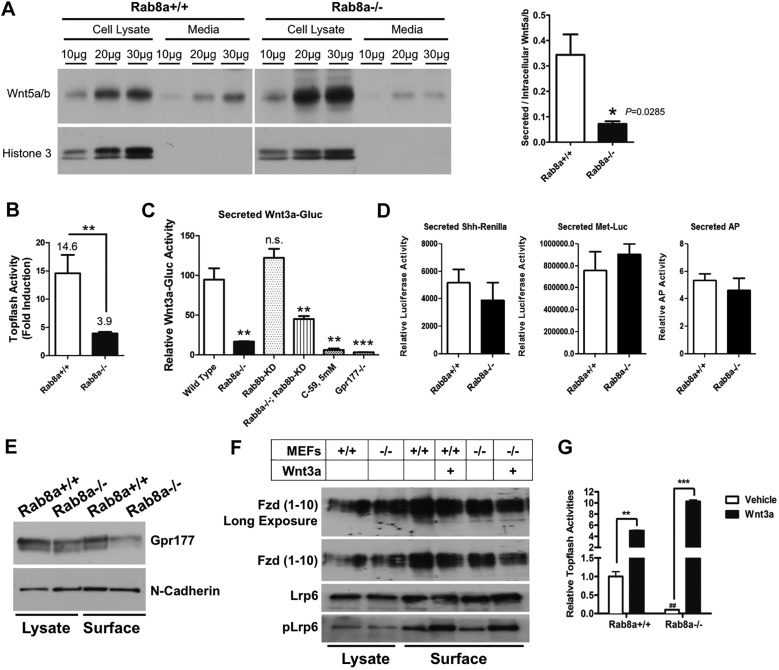
***Rab8a* deletion reduces Wnt secretion.** (A) Concentrated conditioned media or cell lysates, in increasing amounts, from *Rab8a^+/+^* and *Rab8a^−/−^* MEFs were analyzed by western blot. *Rab8a^−/−^* MEFs showed less secreted, but more intracellular, Wnt5a/b. The ratio of secreted to intracellular Wnt5a/b was deduced from corresponding samples (*n*=3, bar chart). Histone H3 was used to detect medium contamination by cell lysates and to normalize corresponding Wnt5a/b bands. (B) WNT5A expression constructs or empty vectors were transiently transfected together with Topflash reporter and Renilla luciferase plasmids into *Rab8a^+/+^* and *Rab8a^−/−^* MEFs, followed by dual-luciferase assays. *Rab8a^+/+^* and *Rab8a^−/−^* MEFs showed 14.6-fold and 3.9-fold inductions of Topflash activities, respectively, by transfected WNT5A as compared with empty vector-transfected counterparts. **P<0.01. (C) *Rab8a^−/−^* MEFs secrete less Wnt3a-Gluc. Wnt3a-Gluc was transiently transfected into wild-type, *Rab8a^−/−^*, Rab8b knockdown (KD), *Rab8a^−/−^/*Rab8b KD and *Gpr177^−/−^* MEFs, with firefly luciferase serving as control for transfection efficiency. Secretion of Wnt3a-Gluc was eliminated by 5 mM C59 or *Gpr177* depletion. **P<0.01, ***P<0.001; n.s., not significant. (D) *Rab8a^−/−^* MEFs showed insignificant changes in the secretion of Shh-Renilla, Met-Luc or the biosynthetic cargo alkaline phosphatase (AP). (E) Surface protein biotinylation and isolation detected decreased Gpr177 in *Rab8a^−/−^* MEFs. Consistent results were obtained in two independent experiments. (F) *Rab8a^−/−^* MEFs showed similar levels of cell surface Fzd (1-10) and Lrp6 compared with *Rab8a^+/+^* MEFs. Note that Wnt3a stimulated surface Lrp6 phosphorylation (Ser1490) in *Rab8a^+/+^* and *Rab8a^−/−^* MEFs. (G) Topflash assays showed that *Rab8a^−/−^* MEFs, with a significantly lower basal Wnt signaling activity (^##^*P*<0.01, compared with vehicle-treated *Rab8a^+/+^* MEFs), responded strongly to Wnt3a stimulation. **P<0.01, ***P<0.001, compared with vehicle-treated cells.

Impaired Gpr177 traffic might influence its function in Wnt exocytosis. As Wnt5a is endogenously expressed by MEFs (Sato et al., 2004) and is known to influence non-canonical and canonical Wnt pathways in mammalian cells (He et al., 1997; Mikels and Nusse, 2006; Okamoto et al., 2014), we compared Wnt5a/b secretion in *Rab8a^+/+^* and *Rab8a^−/−^* MEFs. Using a Wnt5a/b-specific antibody, we detected significant reductions in secreted Wnt5a/b in media conditioned by *Rab8a^−/−^* MEFs (Fig. 2A). *Rab8a^−/−^* MEFs accumulated more intracellular Wnt5a/b, as indicated in cell lysates (Fig. 2A), suggesting that a blockage of Wnt5a/b secretion might have caused ligand accumulation. When exogenous WNT5A was transiently transfected into *Rab8a^+/+^* MEFs, it enhanced Wnt Topflash reporter activity (Fig. 2B), suggesting elevated WNT5A autocrine signaling (Goel et al., 2012). However, similar overexpression of WNT5A in *Rab8a^−/−^* MEFs failed to augment Wnt reporter activity (Fig. 2B), suggesting that *Rab8a^−/−^* MEFs might not properly secrete the transfected WNT5A.

We examined whether Rab8a deficiency also impaired secretion of canonical Wnt ligands such as Wnt3a. MEFs do not detectably express endogenous Wnt3a. Wnt3a-Gluc, a fusion protein comprising Wnt3a and Gaussia luciferase (Chen et al., 2009), has been shown to act as a functional ligand and has been successfully used to screen for small molecular inhibitors of Porcupine (Chen et al., 2009). We transiently transfected *Rab8a^+/+^* and *Rab8a^−/−^* MEFs with Wnt3a-Gluc and assessed the conditioned media by luciferase assay (Chen et al., 2009). We detected an ∼80% reduction of secreted Wnt3a-Gluc in *Rab8a^−/−^* MEF-conditioned media as compared with *Rab8a^+/+^* MEF-conditioned media (Fig. 2C). No reduction of Wnt3a-Gluc secretion was detected for MEFs with stable *Rab8b* depletion by a lentiviral shRNA against *Rab8b* (Fig. 2C). Combined depletion of *Rab8a* and *Rab8b* did not elicit additive inhibition of Wnt3a-Gluc secretion as compared with *Rab8a* deletion alone (Fig. 2C), suggesting that Rab8b, neither by itself nor in combination with Rab8a, influences Wnt3a-Gluc secretion. This is in agreement with the primary function of RAB8B in ligand-receiving cells (Demir et al., 2013). Stronger inhibitory effects on Wnt3a-Gluc secretion were observed upon treatment with C59 (∼93% reduction), a Porcupine inhibitor (Chen et al., 2009), or by *Gpr177* ablation in MEFs (∼97% reduction) (Fig. 2C). Notably, in contrast to the near-complete loss of Wnt3a-Gluc secretion caused by C59 treatment or *Gpr177* deletion, 19% of Wnt3a-Gluc proteins were still detected in *Rab8a^−/−^* MEF-conditioned media (Fig. 2C), suggesting that *Rab8a* deletion partially compromised Wnt secretion, observations that are consistent with the data on Wnt5a/b secretion (Fig. 2A). We further examined the secretory activities of several non-Wnt ligands, including sonic hedgehog (Shh)-Renilla luciferase (Ma et al., 2002) as another important morphogen, the biosynthetic cargo secreted alkaline phosphatase (Yu et al., 2014c) and the constitutively secreted cargo Metridia luciferase (Markova et al., 2004). None of these showed significant secretory abnormalities in *Rab8a^−/−^* MEFs (Fig. 2D), suggesting a degree of cargo selectivity by Rab8a.

### *Rab8a* deletion reduces anterograde transport of Gpr177

Rab8a vesicles control the apical transport of brush border enzymes in enterocytes (Sato et al., 2014, 2007) and are responsible for transporting cargoes for exocytosis (Khandelwal et al., 2013; Sato et al., 2007; Sun et al., 2014). To test whether Rab8a is necessary for anterograde transport of Gpr177 to the cell surface, we directly measured surface-localized Gpr177 by surface-protein biotinylation and isolation. We detected an ∼46% reduction of surface Gpr177 in *Rab8a^−/−^* MEFs (Fig. 2E). In contrast to Gpr177, the amount of surface-localized Fzd receptors and Lrp6 co-receptor did not change (Fig. 2F). Most importantly, addition of Wnt3a proteins to cultured *Rab8a^−/−^* MEFs markedly stimulated cell surface Lrp6 phosphorylation (Ser1490) to a level equivalent to that of *Rab8a^+/+^* cells (Fig. 2F), suggesting that *Rab8a^−/−^* cells can properly respond to exogenous ligand stimulation by assembling an Lrp6-containing surface protein complex (Bilic et al., 2007). In Topflash reporter assays, *Rab8a^−/−^* MEFs showed lower basal reporter activities in serum-deprived conditions (white bars in Fig. 2G), and again responded strongly to exogenous Wnt3a (Fig. 2G).

### Reduced Wnt/β-catenin signaling in *Rab8a^−/−^* intestinal crypts

**Fig. 3.**
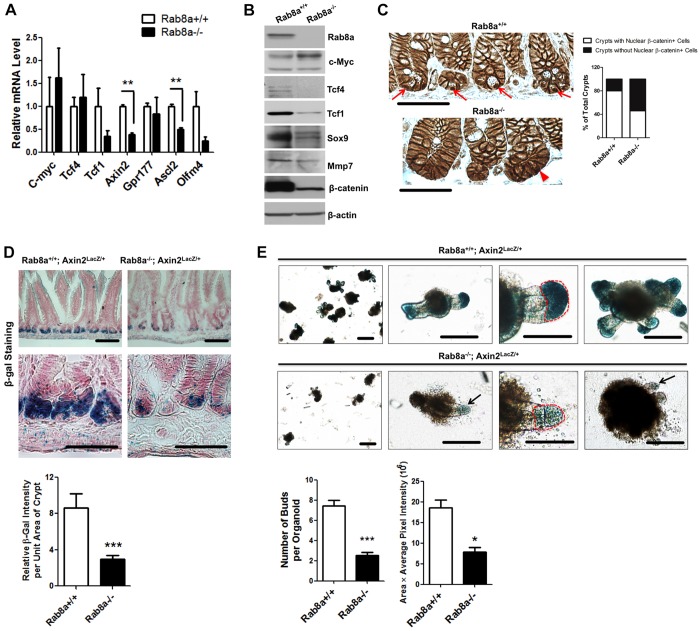
***Rab8a* deletion impairs canonical Wnt signaling in intestines.** (A) Quantitative RT-PCR showed reduced *Tcf1*, *Olfm4*, *Axin2* and *Ascl2* expression in *Rab8a^−/−^* intestines. (B) Western blots showed reduced Tcf1, Tcf4, Sox9 and β-catenin levels in *Rab8a^−/−^* intestines. (C) Immunohistochemistry for β-catenin showed a reduced number of crypts with nuclear β-catenin^+^ cells (arrows). Note that only crypts with detectable Paneth cells were scored for their positive or negative inclusion of nuclear β-catenin (*n*=50 for each genotype). (D) β-Gal staining of mouse small intestines showed a significant reduction of Axin2 reporter activity in crypts in *Axin2^lacZ/+^;Rab8a^−/−^* mice. Thirty continuous crypts were analyzed in each section of independent wild-type and knockout mice. (E) β-Gal-stained *Axin2^lacZ/+^* and *Axin2^lacZ/+^;Rab8a^−/−^* intestinal organoids showed significantly reduced bud number and size in the absence of Rab8a. Images were taken at day 10 after crypt plating. Arrow points to a small bud. Twenty organoids of each genotype were quantified for β-gal-stained bud areas (circled in red). *P<0.05, **P<0.01, ***P<0.001. Scale bars: 10 μm in C,D; 15 μm in E.

Intestinal crypt homeostasis relies on proper Wnt signaling in the stem cell niche (Clarke, 2006; Gregorieff and Clevers, 2005; Haegebarth and Clevers, 2009). Quantitative RT-PCR for canonical Wnt targets showed decreased *Axin2* and *Ascl2* expression in *Rab8a*^−/−^ intestines (Fig. 3A). Western blots detected reduced levels of total β-catenin, Tcf1 (T-cell factor 1; also known as Tcf7), Tcf4 and Sox9 (Fig. 3B). However, the level of c-Myc, a marker of transit amplifying cells (Gregorieff et al., 2005), increased in *Rab8a*^−/−^ intestines (Fig. 3B). Immunohistochemical analyses of β-catenin showed a reduced number of crypts with nuclear β-catenin^+^ cells (only Paneth cell-containing crypts were compared, Fig. 3C). Within a single *Rab8a^−/−^* crypt, the number of nuclear β-catenin^+^ cells was also reduced compared with wild types, collectively suggesting reduced Wnt/β-catenin signaling in knockout crypts.

We then used the *Axin2^l^^acZ/+^* reporter allele to determine canonical Wnt signaling activity (Lustig et al., 2002). By establishing *Rab8a^−/−^;Axin2^lacZ/+^* mice, we observed reduced *Axin2^lacZ/+^* reporter activities, as indicated by β-gal staining of mouse small intestines, compared with *Rab8a^+/+^;Axin2^lacZ/+^* littermates (Fig. 3D).

**Fig. 4.**
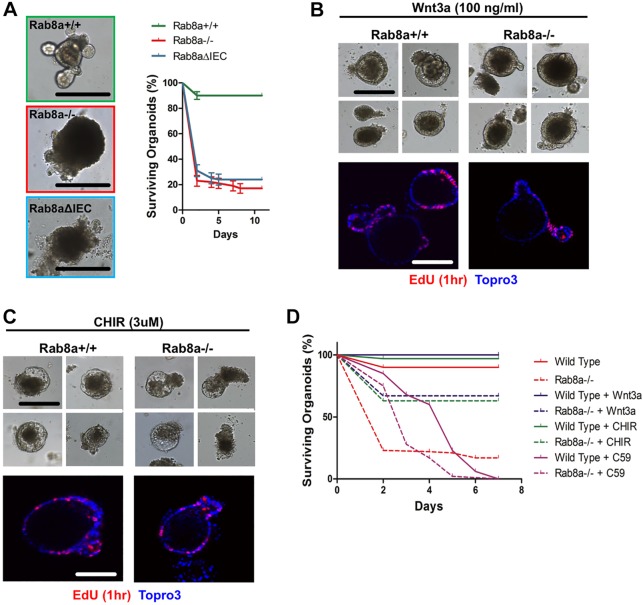
**Rab8a deficiency-impaired organoid growth is restored by Wnt3a.** (A) Crypts (*n*=100) of each genotype were seeded in triplicate and the number of surviving organoids then counted daily. Growth of 80% of *Rab8a^−/−^* intestinal organoids was arrested within the first 2 days. *Rab8a*^Δ*IEC*^ did not show any significant improvement in organoid survival. Data were collected from three independent experiments. (B,C) After seeding, *Rab8^+/+^* and *Rab8a^−/−^* intestinal organoids (*n*=100 for each genotype) were immediately supplemented with Wnt3a or the Gsk3β inhibitor CHIR at the indicated concentrations. Media containing these supplements were replenished daily. *Rab8a^−/−^* intestinal organoids showed similar morphological and proliferative features as *Rab8^+/+^* organoids upon Wnt3a or CHIR treatment. (D) In the above experiments, surviving organoids were counted daily during 1 week after seeding. Percentages of surviving organoids are plotted. Note that Wnt3a and CHIR significantly increased the number of surviving *Rab8a^−/−^* organoids. Scale bars: 15 μm.

We further analyzed *Axin2^lacZ/+^* reporter activity in cultured intestinal organoids. *Rab8a^−/−^;Axin2^lacZ/+^* organoids also showed reduced β-gal activities in epithelial buds when compared with those in *Rab8a^+/+^;Axin2^lacZ/+^* epithelial buds (Fig. 3E). Remarkably, *Rab8a^−/−^* intestinal organoids, as well as *Rab8a^loxP/loxP^;Villin-Cre* (*Rab8a^ΔIEC^*) organoids, showed severely compromised growth and budding capability, with nearly 80% arrested within 2 days (Fig. 4A). The majority of surviving *Rab8a^−/−^* organoids showed tiny buds and large lumens (Fig. 3E and Fig. 4A), similar to Wnt3*-* or Atoh1*-*deficient enteroids (Durand et al., 2012; Farin et al., 2012). Administration of the Porcupine inhibitor C59 to surviving *Rab8a^−/−^* organoids further abolished epithelial budding, followed by the near-complete disappearance of existing buds within 4 days of treatment (101 out of 105 Rab8a-deficient organoids; supplementary material Fig. S2A). A fraction of C59-treated organoids maintained cyst-like structures; however, their proliferative activities were markedly reduced as assessed by 5-ethynyl-2′-deoxyuridine (EdU) labeling (supplementary material Fig. S2B). C59 treatment presumably depleted residual Wnt secretion from Rab8a*-*deficient organoids, suggesting that *Rab8a* ablation partially, but not completely, blocked Wnt secretion.

Importantly, when we supplemented culture media with exogenous Wnt3a, the survival and propagation capacities of Rab8a-deficient organoids were significantly improved by ∼50% (Fig. 4B,D). Wnt3a administration induced *Rab8a^+/+^* organoids into a cyst-like morphology (Yin et al., 2014), an effect that was also observed for Rab8a-deficient organoids. Treating Rab8a-deficient organoids with CHIR99021, a Gsk3β inhibitor, improved survival by 46% (Fig. 4C,D), supporting the notion that the poor growth of *Rab8a^−/−^* organoids was due to insufficient Wnts and that Rab8a-deficient cells can properly transduce the Wnt signal.

### Paneth cell maturation is severely impaired in *Rab8a^−/−^* mice

**Fig. 5.**
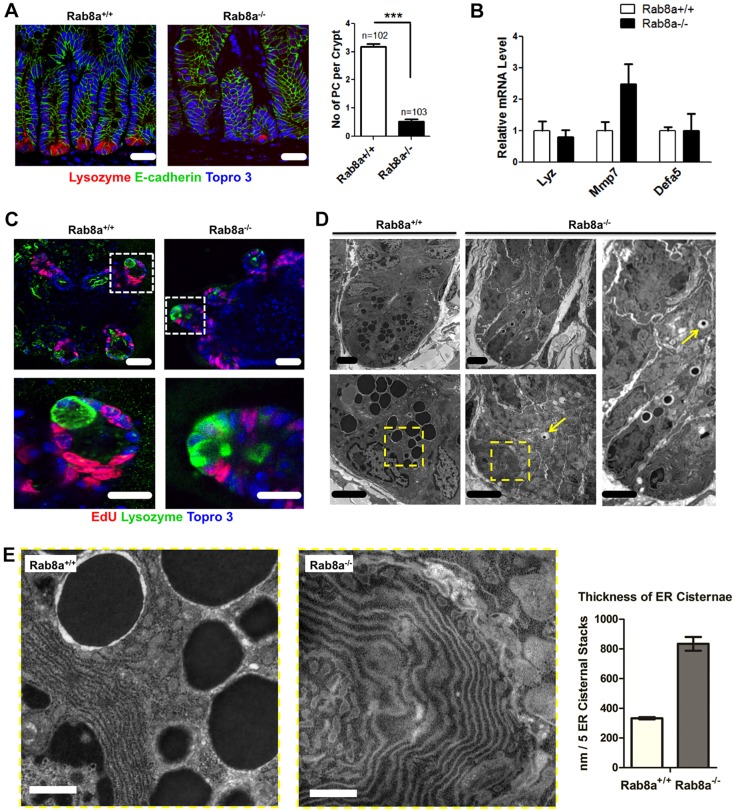
***Rab8a* deletion impairs Paneth cell maturation.** (A) Lysozyme staining showed reduced numbers of lysozyme^+^ Paneth cells in *Rab8a^−/−^* crypts. ***P<0.001. (B) Quantitative RT-PCR showed no significant change in the Paneth cell gene expression signature in *Rab8a^−/−^* intestinal tissues. (C) Lysozyme and EdU (1 h) staining of *Rab8a*^−/−^ organoids detected lysozyme^+^ Paneth cells in proliferating buds of surviving organoids. (D) TEM showed that the residual Paneth cells in *Rab8a^−/−^* crypts exhibit a reduction in the typical number and size of granules. Arrow points to a granule in a *Rab8a^−/−^* crypt. (E) An expansion of smooth ER was observed in all remaining *Rab8a^−/−^* Paneth cells. The thickness of stacked ER cisternae was significantly expanded in *Rab8a^−/−^* (834±46 nm) as compared with *Rab8a^+/+^* (333±7 nm) Paneth cells. Scale bars: 10 µm in A; 15 µm in C; 2 µm in D; 1 µm in E.

Paneth cells are the major epithelial Wnt producers within the intestinal crypts (Sato et al., 2011). Lysozyme staining identified significant reduction of lysozyme^+^ Paneth cells in *Rab8a^−/−^* crypts. Eighty-five percent of *Rab8a^−/−^* intestinal crypts contained virtually no detectable lysozyme^+^ Paneth cells, while the rest had a single lysozyme^+^ cell (Fig. 5A). The decrease in mature lysozyme^+^ Paneth cells re-established the notion that the maturation of this cell type depends on proper Wnt signaling (van Es et al., 2005). Interestingly, when compared with *Rab8a^+/+^*, *Rab8a^−/−^* intestines transcribed similar or even higher levels of several Paneth cell-specific genes, namely *Lyz1*, *Mmp7* and *Defa5* (Fig. 5B), suggesting that *Rab8a* deletion might have actually blocked the terminal differentiation of Paneth cells from the precursors. Such intermediate secretory precursors have recently been described (Buczacki et al., 2013; Clevers et al., 2014). In cultured organoids, lysozyme staining identified Paneth cells in small buds of surviving *Rab8a^−/−^* organoids (green, Fig. 5C), hinting that the preservation of a small number of Paneth cells in surviving knockout crypts might have facilitated their survival, whereas crypts totally lacking mature Paneth cells were arrested (red line, Fig. 4A).

Analyses of *Rab8a^−/−^* intestinal crypts by transmission electron microscopy (TEM) revealed substantial subcellular defects in these residual Paneth cells. Virtually all *Rab8a^−/−^* Paneth cells contained fewer electron-dense secretory granules than typically found in this cell type (Fig. 5D). *Rab8a^−/−^* Paneth cells also showed markedly expanded (∼2.5-fold) smooth ER cisternal stacks as compared with those in *Rab8a^+/+^* Paneth cells (Fig. 5E; supplementary material Figs S3 and S4).

Various non-Paneth cells surrounding crypts produce Wnts, and thus redundant sources of Wnts have been proposed to support the stem cell niche (Farin et al., 2012; Gregorieff et al., 2005). *Rab8a* global knockout (*Rab8a^−/−^*) presumably affected Wnt secretion from all niche supporting cells. We postulated that intestinal epithelial cell-specific *Rab8a* deletion (*Rab8a*^Δ*IEC*^) would produce a milder phenotype than that of *Rab8a^−/−^* intestines. Indeed, compared with *Rab8a^−/−^* intestines, *Rab8a^ΔIEC^* intestines exhibited less pronounced Paneth cell loss. Continuous stretches of *Rab8a^ΔIEC^* crypts containing Paneth cells existed next to crypts without Paneth cells, suggesting an overall milder impact on Paneth cell maturation (supplementary material Fig. S5A,B). This observation was echoed in organoid culture experiments. We observed two morphologically distinct clones of *Rab8a^ΔIEC^* organoids: one resembled wild type (supplementary material Fig. S5C) with well-developed buds, whereas the other mimicked *Rab8a^−/−^* organoids (Fig. 4A). The visibly improved epithelial budding in a fraction of *Rab8a^ΔIEC^* organoids agreed with the observation that some *Rab8a^ΔIEC^* crypts possessed more Paneth cells than others. Nevertheless, *Rab8a^ΔIEC^* did not show improved long-term survival compared with *Rab8a^−/−^* organoids (compare blue and red lines, Fig. 4A).

### *Rab8a* deletion decreases Gpr177 trafficking to the cell surface *in vivo*

**Fig. 6.**
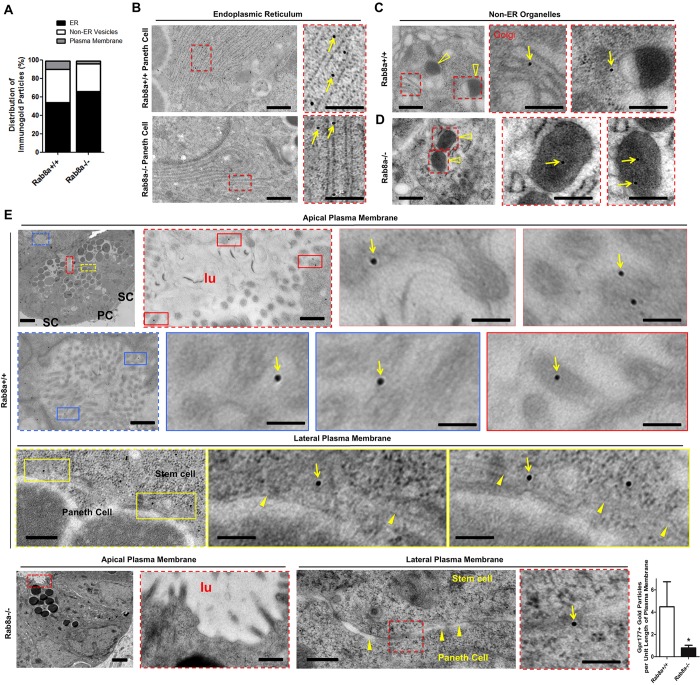
***Rab8a* deletion affects Gpr177 transport to the plasma membrane but not ER export.** (A) Quantification of Gpr177^+^ immunogold particle distributions in the ER, non-ER Golgi/vesicle and plasma membrane of *Rab8a^+/+^* and *Rab8a^−/−^* Paneth cells (particles counted from 12 *Rab8a^+/+^* and nine *Rab8a^−/−^* Paneth cells from three *Rab8a^+/+^* and two *Rab8a^−/−^* mice, respectively). (B) The majority of Gpr177^+^ immunogold particles (arrows) were detected in the ER of *Rab8a^+/+^* and *Rab8a^−/−^* Paneth cells. (C,D) Representative micrographs showing Gpr177^+^ immunogold particles in Golgi and lysosomes. Gold particles were detected in lysosomes (open arrowheads) in *Rab8a^−/−^* (D) but not in *Rab8a^+/+^* (C). (E) Gpr177^+^ immunogold particles were frequently detected at apical or basolateral plasma membranes in wild-type Paneth cells. A significant reduction in plasma membrane-localized Gpr177^+^ particles was detected in *Rab8a^−/−^* Paneth cells per unit length of plasma membrane. Data were collected for a total length of 312 µm plasma membrane in 12 *Rab8a^+/+^* Paneth cells and 328 µm plasma membrane in 14 *Rab8a^−/−^* Paneth cells from three *Rab8a^+/+^* and two *Rab8a^−/−^* mice, respectively. Arrowheads point to plasma membranes between a stem cell and a Paneth cell. lu, lumen. *P<0.05. Scale bars: 500 nm.

To further explore whether Gpr177 traffic was affected in Wnt producers *in vivo*, we performed immunogold labeling of Gpr177 in *Rab8a^+/+^* and *Rab8a^−/−^* mouse intestines. The specificity of the Gpr177 antibody (Fu et al., 2009) was affirmed by a 90%, 91% and 93% reduction in gold particles in the ER, the non-ER vesicular compartment and the plasma membrane, respectively, of Gpr177-deficient cells (supplementary material Fig. S6) as compared with wild-type cells. Using identical labeling conditions, we detected an 11% total reduction of Gpr177^+^ gold particles in *Rab8a^−/−^* Paneth cells. From TEM montage images we performed quantitative analyses of gold particle distributions at the ER, plasma membrane and non-ER Golgi/secretory vesicular compartments (independent duodenal and jejunum segments from three *Rab8a*^+/+^ and two *Rab8a*^−/−^ mice, Fig. 6A-D). A large portion of the Paneth cell cytoplasm is occupied by ER, where the majority of Gpr177^+^ gold particles were detected (Fig. 6A,B). Comparison of the intracellular distribution of gold particles in *Rab8a^+/+^* and *Rab8a^−/−^* Paneth cells indicated statistically insignificant differences in Gpr177 distribution in ER or non-ER compartments (Fig. 6A), suggesting that Gpr177 can be exported from the ER without Rab8a. However, we identified a significant reduction of Gpr177^+^ gold particles adjacent to apical or basolateral plasma membranes (Fig. 6E; more *Rab8a^+/+^* and *Rab8a^−/−^* cells are shown in supplementary material Figs S7 and S8 respectively). This reduced peripheral Gpr177 localization was reflected in fewer surface gold particles per Paneth cell, as well as in an ∼88% reduction of particles per unit length of Paneth cell plasma membrane (Fig. 6E). A similar trend was observed in *Rab8a^−/−^* subepithelial stromal cells (supplementary material Fig. S9), which agreed with data from the biochemical isolation of surface Gpr177 (Fig. 2E).

Given that Gpr177 is able to exit the ER, we postulated that Rab8a absence might lead to mis-sorting of Gpr177 into endolysosomal compartments, an observation made for microvillus enzymes in *Rab8a^−/−^* enterocytes (Sato et al., 2007). We were able to examine lysosomes and multivesicular bodies (MVBs) owing to their distinctive morphologies (Fig. 6C,D; supplementary material Fig. S10). We detected frequent localization of Gpr177^+^ gold particles to lysosomes and MVBs in *Rab8a^−/−^* but not *Rab8a^+/+^* cells (Fig. 6D; supplementary material Fig. S10), suggesting that the absence of Rab8a might have caused endolysosomal targeting of Gpr177.

Western blots for endogenous Gpr177 using intestinal lysates showed multiple protein species, reflecting previously reported post-translational modification and alternative splicing events (Tanaka et al., 2002; Yu et al., 2010, 2014a). *Rab8a^−/−^* intestines showed a reduction of the 62 kDa and 53 kDa protein species (full-length Gpr177 is predicted to be 62 kDa) (lanes 1-6, supplementary material Fig. S11A). *Rab8a*^Δ*IEC*^ intestines, with intact Rab8a in non-epithelial cells, retained the 53 kDa Gpr177 isoform (lanes 7-8, supplementary material Fig. S11A), which was present in wild-type MEFs but not *Gpr177*^−/−^ MEFs (lanes 9-10, supplementary material Fig. S11A). Although these data clearly demonstrated a certain impact of Rab8a loss on Gpr177 protein patterns, the mixed cell population in a tissue context appeared to complicate the interpretation.

Thus, we directly determined whether Rab8a loss increases endolysosomal transport of Gpr177 by establishing a Caco2 cell line with stable RAB8A knockdown. Compared with Caco2 cells treated with non-specific shRNA, RAB8A depletion caused a 3-fold increase in targeting of GPR177-EGFP into LAMP1^+^ (a lysosomal membrane protein) compartments (supplementary material Fig. S11B). Treatment of RAB8A-depleted Caco2 cells with bafilomycin A, which blocks endolysosomal functions, increased total GPR177 levels (supplementary material Fig. S12). Conversely, when protein synthesis was blocked by treating RAB8A-depleted Caco2 cells with cycloheximide, a faster reduction in the GPR177 level was observed when compared with cycloheximide-treated control cells (supplementary material Fig. S13). These data suggested that, in the absence of RAB8A-mediated exocytosis, there is an enhanced endolysosomal transport and clearance of GPR177, yet RAB8A-depleted cells continue to make new GPR177.

### *Rab8a* deletion induces changes of crypt cell organization

**Fig. 7.**
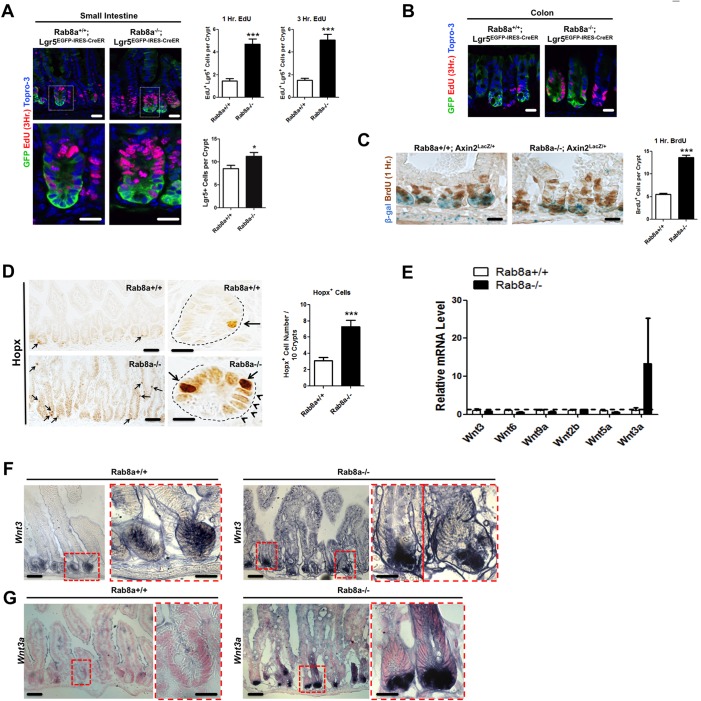
**Adaptive changes in *Rab8a^−/−^* crypts.** (A) EdU labeling (1 and 3 h, red) of mouse small intestine showed a significant increase in proliferative Lgr5^+^ cells in *Rab8a^−/−^;Lgr5^EGFP−IRES−CreERT2^* mice. The total number of Lgr5^+^ cells was also increased in *Rab8a^−/−^* crypts. Around 45 crypts that contained Lgr5^+^ cells were analyzed in each tissue section of independent *Lgr5^EGFP−IRES−CreERT2^* or *Rab8a^−/−^;Lgr5^EGFP−IRES−CreERT2^* mice. (B) EdU labeling of the colon showed an increase in proliferative Lgr5^+^ cells in *Rab8a^−/−^;Lgr5^EGFP−IRES−CreERT2^* mice. (C) Co-staining of BrdU (1 h, brown) and β-gal (blue) showed increased BrdU^+^ cells in *Axin2^l^^acZ/+^;Rab8a^−/−^* intestines, despite decreased Axin2 reporter activity in the same crypts. One hundred continuous crypts were analyzed in each section of independent *Rab8a^+/+^* and *Rab8a^−/−^* mice. (D) *Rab8a^−/−^* intestines contained more cells with the strongest level of Hopx immunoreactivity (arrows). Arrowheads point to cells with moderately higher immunoreactivity than in wild type. One hundred continuous crypts were analyzed in each section of independent *Rab8a^+/+^* and *Rab8a^−/−^* mice. (E) Quantitative RT-PCR detected increased *Wnt3a* levels in *Rab8a*^−/−^ intestines (*n*=3 for each genotype). (F,G) RNA *in situ* hybridization to detect *Wnt3* (F) or *Wnt3a* (G), showing ectopic activation of *Wnt3a* in *Rab8a^−/−^* crypts, whereas *Wnt3* was largely unaffected. *P<0.05, ***P<0.001. Scale bars: 10 µm.

Rab8a-deficient mice provided an opportunity to examine the crypt response to weakened Wnt production at the stem cell niche. We first examined the fast-cycling crypt base columnar (CBC) cells in *Rab8a^−/−^* mice using Lgr5 as an indicator (Barker et al., 2007). We derived *Rab8a^−/−^;Lgr5^EGFP−IRES−CreERT2/+^* mice, and analyzed proliferative Lgr5^+^ stem cells by EGFP and EdU labeling. Increased EdU^+^ Lgr5^+^ cells were detected in both the small intestine and colon of *Rab8a^−/−^;Lgr5^EGFP−IRES−CreERT2/+^* mice (Fig. 7A). Total numbers of Lgr5^+^ cells per crypt also increased in *Rab8a^−/−^* intestines (Fig. 7A). The numbers of transit amplifying cells and mitotic crypt cells (phosphorylated histone H3^+^) were also increased in *Rab8a^−/−^* crypts (supplementary material Fig. S14; data not shown), which might explain the increased c-Myc in *Rab8a^−/−^* intestines (Fig. 3B). Co-staining for β-gal and BrdU using *Rab8a^−/−^;Axin2^l^^acZ/+^* tissues identified a 2-fold increase in BrdU^+^ cells in *Rab8a^−/−^* crypts, where reduced β-gal activity was seen (Fig. 7C). Thus, the *in vivo* proliferation of *Rab8a^−/^*^−^ crypt cells did not appear to be impacted by weakened Wnt signaling – an unexpected observation that contrasts with the drastic loss of *Rab8a^−/−^* organoids *in vitro* (see Discussion).

Crypt cells demonstrate great plasticity (Tetteh et al., 2014). The tissue regenerative program could be activated by injury in a number of slow-cycling or secretory precursor cell types (Buczacki et al., 2013; Powell et al., 2012; Tian et al., 2011; van Es et al., 2012; Yan et al., 2012). Bmi1^+^ cells have been shown to be relatively resistant to Wnt signaling perturbation (Yan et al., 2012). The reduced Wnt concentration in *Rab8a^−/−^* crypts might stress crypt cells into certain adaptive changes. Using quantitative RT-PCR we surveyed several ‘quiescent’ stem cell markers but only detected an increase in the *Bmi1* mRNA level (supplementary material Fig. S15). Using a Hopx antibody, we also analyzed the reported +4 cell type that has been shown by lineage tracing to convert to Lgr5^+^ cells (Takeda et al., 2011). *Rab8a^+/+^* intestinal epithelia contained about three Hopx^+^ cells (experimentally defined as strongly immunopositive for nuclear Hopx) in every ten adjacent crypts, whereas *Rab8a^−/−^* intestines showed about seven or eight Hopx^+^ cells in the same number of crypts (Fig. 7D). *Rab8a^−/−^* intestines also contained multiple Hopx^+^ cells in a single crypt, with generally stronger nuclear immunoreactivity compared with *Rab8a^+/+^* intestines (Fig. 7D, arrows and arrowheads indicate strong and moderate staining, respectively). Hopx^+^ cells were found in *Rab8a^−/−^* epithelium at positions other than +4 (Fig. 7D). One-hour EdU labeling of *Rab8a^−/−^* mice led to positive labeling of some Hopx^+^ cells, whereas this was rarely found in *Rab8a^+/+^* crypts (supplementary material Fig. S16A). Co-immunofluorescence analyses of Lgr5^+^ cells and Hopx^+^ cells in *Rab8a^−/−^;Lgr5^EGFP−IRES−CreERT2/+^* intestines showed increased numbers of both cell types (supplementary material Fig. S16B).

*Wnt3*, but not *Wnt3a*, is normally expressed in Paneth cells of the adult mouse intestine (Farin et al., 2012; Gregorieff et al., 2005). When we conducted Wnt ligand expression analyses, we unexpectedly detected ectopic *Wnt3a* mRNA in *Rab8a^−/−^* intestinal crypts (Fig. 7E). By *in situ* hybridization with *Wnt3*- and *Wnt3a*-specific probes (Roelink and Nusse, 1991), ectopic activation of *Wnt3a* was confirmed in *Rab8a^−/−^* crypts (Fig. 7G), whereas *Wnt3* was largely unaffected (Fig. 7F). This ectopic *Wnt3a* expression might suggest a cell type change rather than increased Wnt3a secretion from these cells, as *Rab8a^−/−^* cells failed to properly secrete Wnt3a-Gluc (Fig. 2C). Together, these data suggested that, in the absence of Rab8a, the weakened Wnt signaling blocked Paneth cell maturation from Lgr5^+^ secretory precursors and that this might have initiated an aberrant differentiation program.

## DISCUSSION

Our study of Rab8a function in the mouse intestinal crypt compartment extended previous analyses performed in differentiated enterocytes (Sato et al., 2007). The crypt defects observed in *Rab8a* knockout mice are likely to precede the apical transport abnormalities in enterocytes that are derived from the crypt progenitors. Our finding that Rab8a affects Wnt secretion and Paneth cell maturation impinges on a growing body of evidence linking vesicular traffic to niche signal transduction and maintenance (Feng and Gao, 2014; Goldenring, 2013).

### Wnt secretion

*Rab8a* deletion clearly impacted Wnt signaling in the intestinal crypts. This was strongly supported by *Axin2* reporter analysis *in vivo* and in organoid culture, as well as by the defective Paneth cell maturation in *Rab8a^−/−^* crypts. Mechanistically, these phenotypes could be caused by defective traffic of Wnt receptors such as Fzd or Lrp6. This possibility was ruled out on the basis of several lines of evidence. First, *Rab8a^−/−^* MEFs still responded to exogenous Wnt ligands by phosphorylating the Lrp6 cytosolic tail and activating a Topflash Wnt reporter. Second, exogenous Wnt3a and Gsk3β inhibitor partially restored the growth and survival of *Rab8a^−/−^* organoids. Third, an independent screening assay by Demir et al. showed that RAB8A does not affect Wnt reception (Demir et al., 2013). These data corroborated the view that, unlike RAB8B which regulates Wnt reception and LRP6 endocytosis, RAB8A plays a distinct role in Wnt-producing cells. Accordingly, we did not observe an impact of RAB8B on Wnt secretion.

**Fig. 8.**
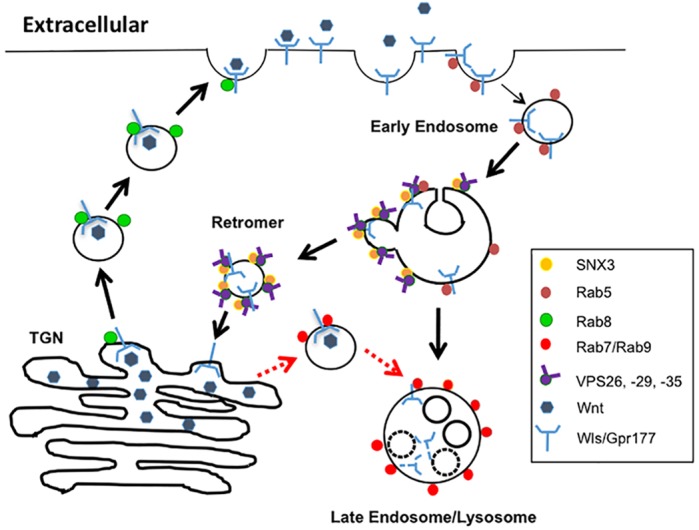
**Rab8a facilitates anterograde transport of Gpr177-Wnt.** Rab8a facilitates the transport of post-Golgi Gpr177-Wnt vesicles to the plasma membrane for secretion. Loss of Rab8a attenuates Gpr177 exocytotic traffic and, via an unknown mechanism (red dotted arrows), may reroute Gpr177 into endolysosomes. TGN, trans-Golgi network.

In *Rab8a^−/−^* Paneth cells or subepithelial fibroblasts, immunogold labeling of Gpr177 and surface protein isolation demonstrated reduced localization of Gpr177 adjacent to the cell surface. It appears that disruption of Rab8a-dependent transport weakens Gpr177-mediated Wnt secretion by rerouting Gpr177 into endolysosomal compartments (Fig. 8). A similar observation has been made following defective retromer-dependent Gpr177 retrieval (Eaton, 2008). After synthesis in the ER, lysosomal hydrolases and membrane proteins are transported directly from the Golgi apparatus and trans-Golgi network to late endosomes via vesicles that exclude other proteins intended for distinct destinations. Possibly, loss of Rab8a causes mispackaging of Gpr177 into lysosome-targeting vesicles. However, it is still unclear how loss of Rab8a would trigger Gpr177 vesicular flow into lysosome-targeting vesicles. In the absence of Rab8a, post-Golgi Gpr177 vesicles might be hijacked by a distinct Rab GTPase (e.g. Rab9), facilitating endolysosomal fusion. Brush border enzymes are also mistargeted into lysosomes in *Rab8a^−/−^* enterocytes (Sato et al., 2007), hinting at some similarities in defective cargo transport in the absence of Rab8a. In different cell types we observed differential influences of Rab8a loss on the Gpr177 protein pattern. This was probably attributable to cell-specific trafficking factors. Thus, the molecular basis of Rab8a-mediated Gpr177 traffic remains to be explored in detail. Of note, Sec4, the Rab8a homolog in yeast, predominantly mediates post-Golgi vesicle secretion (Das and Guo, 2011; Hutagalung and Novick, 2011; Seabra et al., 2002).

The view that Rab8a vesicles promote the export of Gpr177-Wnt also provided additional support for the physiological involvement of Rab8 in GPCR anterograde trafficking (Wang and Wu, 2012). RAB8 was shown to modulate the exocytosis of metabotrophic glutamate receptor (Esseltine et al., 2012) and α2B and β2 adrenergic receptors (Dong et al., 2010) in human embryonic kidney cells and primary neurons, with an interaction with the C-terminus of β2 adrenergic receptors (Dong et al., 2010).

In *Rab8a^−/−^* MEFs, we observed a rather selective impact on Wnt secretion. Rabs and other membrane trafficking regulators are often considered to be generic modulators of protein transport. However, accumulating evidence suggests that perturbing these trafficking processes impacts a rather specific pathway (Knowles et al., 2015; Yu et al., 2014c). For instance, depletion of RAB8B impacted Wnt signalosome activity but not other signaling pathways (Demir et al., 2013). Disrupting retromer function by ablating Vps35 or Snx3 impaired primarily Wnt secretion (Eaton, 2008; Harterink et al., 2011). Furthermore, partial loss of Rab1 and Rab11 preferentially affected Notch signaling in *Drosophila* (Charng et al., 2014; Emery et al., 2005), whereas genetic mutation of *Rab23* specifically perturbed the Hedgehog signaling pathway (Eggenschwiler et al., 2001; Evans et al., 2003), with its potential targets being Suppressor of fused (Chi et al., 2012) or/and Smoothened – another GPCR (Boehlke et al., 2010). As individual vesicles contain heterogeneous cargoes, it is unlikely that Rab8a only traffics Gpr177.

### Paneth cells

The organoid-forming capacity of Lgr5^+^ stem cells in culture is enhanced by association with Paneth cells (Sato et al., 2011). We found that *Rab8a^−/−^* organoids resemble *Wnt3^−/−^* or *Atoh1^−/−^* organoids (Durand et al., 2012; Farin et al., 2012), showing poor clonogenic activities. The vast majority of surviving Rab8a organoids contained tiny Paneth cell-containing buds, suggesting that the few remaining Paneth cells facilitated residual clonogenic activities of the knockout organoids. These data favored the notion that Paneth cells constituted the major stem cell supporters in cultured enteroids after growth support from non-epithelial compartments was eliminated. When *Rab8a^−/−^* organoids were treated with Porcupine inhibitor to deplete residual Wnt production, their growth was further arrested, indicating that *Rab8a* deletion did not completely abrogate Wnt secretion.

*In vivo*, most *Rab8a^−/−^* intestinal crypts were largely devoid of lysozyme^+^ Paneth cells. However, the transcriptional level of multiple Paneth cell-specific genes remained normal. This suggested that the maturation of Paneth cells, rather than Paneth cell fate commitment, was affected in *Rab8a^−/−^* crypts. Our model represents an independent genetic setting for the evaluation of the crypt response to Paneth cell defects.

A subset of Lgr5^+^ LRCs was identified to express both CBC and Paneth cell gene signatures (Buczacki et al., 2013) and proposed to constitute precursors of Paneth cells capable of regeneration following injury. Our data suggested that the lack of extracellular Wnts in *Rab8a^−/−^* crypts might resemble injury-like stress that blocked Paneth cell maturation from their precursors. Importantly, despite the lack of mature Paneth cells, *Wnt3*-expressing crypt cells remained in *Rab8a* knockout crypts, in addition to some crypt cells ectopically expressing *Wnt3a*, suggesting that aberrant transdifferentiation might be triggered by stress induced by *Rab8a* deletion.

### Stem cells

This study provided an opportunity to examine the intestinal stem cell response to a perturbed niche Wnt signal. First, Rab8a deficiency reduced Wnt secretion by ∼80%. Cells might respond to this by increasing their sensitivity to the ligand. This was indeed observed in *Rab8a^−/−^* MEFs. Second, LRC secretory precursors express both Lgr5 and Paneth cell gene signatures and contribute to epithelial regeneration upon tissue injury (Buczacki et al., 2013). We found a clear blockage of Paneth cell maturation and an increase in Lgr5^+^ cell number in *Rab8a^−/−^* crypts. These increased *Rab8a^−/−^* Lgr5^+^ cells are certainly different from typical CBCs in healthy animals, and might reflect Paneth cell precursors, as they expressed Paneth cell genes. Third, the proposed quiescent crypt cells, in particular those that are Bmi1^+^, have been shown to be resistant to Wnt perturbation and capable of converting into Lgr5^+^ cells (Tian et al., 2011; Yan et al., 2012). *Rab8a^−/−^* crypts also contained more Hopx^+^ cells, in addition to increased Lgr5^+^ cells, collectively suggesting a certain degree of crypt cell repopulation.

Although intestinal crypt-villus damage was induced in extreme Wnt perturbation models, as exemplified by dickkopf homolog 1 (Dkk1) overexpression (Kuhnert et al., 2004; Pinto et al., 2003) and *Tcf4^−/−^* mice (van de Wetering et al., 2002), removal of Wnt production from a number of Wnt-producing sources has so far been insufficient to perturb crypt stem cells (Farin et al., 2012; Kabiri et al., 2014; San Roman et al., 2014). Factors contributing to intestinal regenerative abilities may arise from multiple sources. Injury-induced apoptotic cells have been shown to be a source of Wnt3 to support regeneration (Galliot, 2013). Autocrine Wnts produced by adult stem cells may constitute their self-niche for renewal (Lim et al., 2013). Finally, crypt cells that are insensitive to either R-spondin or Dkk1 may exist to replenish the epithelia (Yan et al., 2012), presumably in a Wnt-independent fashion. Given that the vast majority of colon cancer cells contain a constitutively active Wnt pathway and thereby do not rely on external Wnts, it is not entirely surprising to observe strong cell resilience to Wnt ligand perturbation. Cell lineage analysis might help us to better understand the cellular adaptation in Rab8a-deficient crypt. Whether Gpr177 regulates Wnt secretion in intestinal stem cells in addition to Paneth cells, as it does in other organs (Jiang et al., 2013; Stefater et al., 2011), requires further study. Taken together, *Rab8a* deletion induces epithelial stress that provokes crypt cell alteration. We conclude that Rab8a affects Wnt secretion and Paneth cell maturation at the intestinal stem cell niche.

## MATERIALS AND METHODS

### Mice and cells

*Rab8a^−/−^*, *Rab8a^ΔIEC^*, *Lgr5^EGFP−IRES−CreERT2^*, *Gpr177^fl/fl^* and *Axin2^l^^acZ/+^* mice have been described previously (Barker et al., 2007; Fu et al., 2011; Lustig et al., 2002; Sato et al., 2007). *Rab8a^−/−^* and *Rab8a^ΔIEC^* mice die at ∼4 weeks; all comparisons were made between littermates of at least three mice for each genotype unless stated otherwise. All experiments were repeated two or three times; only consistent results are presented. Experimental procedures were approved by Rutgers University Institutional Animal Care and Use Committee. Procedures for derivation of *Rab8a^−/−^*, Rab8b knockdown, *Gpr177^−/−^* MEFs and intestinal organoids are detailed in the supplementary material Methods. Phenotypic analyses of cells and tissues have been described previously (Gao et al., 2009; Sakamori et al., 2012; Yu et al., 2014b).

### Gpr177 vesicle tracking

MEFs were transfected with Gpr177-mCherry using Lipofectamine 2000 (Life Technologies) as per manufacturer's specifications. Forty-eight hours after transfection, cells were trypsinized and seeded into a 35 mm glass-bottom dish (MatTek Corporation) and imaged after 24 h. Images were acquired using a Zeiss Axio Observer Z1 equipped with a 100× objective, heated stage and CO_2_ controller. Frames were captured at the maximum possible speed allowing *z*-plane correction using a definite focus system (Zeiss). Data were analyzed using ImageJ (NIH). Images were inverted and vesicles tracked using the MTrackJ plug-in (Meijering et al., 2012). Sixteen Gpr177-mCherry vesicles were tracked in control and *Rab8a*^−/−^ MEFs and data were collected from two independent experiments. Speeds were plotted as frequency distribution (percentage of total steps) with a bin size of 70 nm/s.

### Dual-luciferase assay and fluorescent SEAP assay

To measure Wnt3a-Gluc, ShhNRen and Metridia luciferase secretions, MEFs of various genotypes were transiently co-transfected with Wnt3a-Gluc, ShhN-Ren or Met-Luc, with firefly luciferase serving as transfection control. Twenty-four hours after transfection, supernatants and cell lysates were collected and subjected to dual-luciferase assay (Promega, E1980) using the Glomax multidetection system (Promega). Each reaction consisted of 50 μl medium or 10 μl cell lysate, and 50 μl luciferase assay substrate and 50 μl of Stop&Glo Reagent.

The SEAP assay was performed using the Great EscAPe Fluorescence Detection Kit (Clontech, 631704). 25 μl supernatant or cell lysate was diluted with the same volume of 1×dilution buffer in a 96-well plate, mixed gently on a rotating platform for 5 min at room temperature and incubated at 65°C for 30 min. Samples were then cooled to room temperature; 97 μl assay buffer was added and incubated for 5 min at room temperature; 3 μl 1 mM MUP (substrate) was added and incubated with the sample at room temperature for 1 h in the dark. Fluorescent units were read using the Glomax multidetection system. Luminescence units from Gaussia luciferase, Renilla luciferase and Metridia luciferase were normalized against intracellular firefly luciferase. Assays were repeated three or more times with three to six technical replicates per cell line each time.

### Topflash reporter assay

To compare Wnt3a responsiveness in wild-type, *Rab8a*^−/−^ and Rab8b knockdown MEFs, cells were co-transfected with Topflash and Renilla luciferase for 24 h. The cells were then serum starved for 3 h in Wnt-free DMEM and treated with 20 ng/ml recombinant murine Wnt3a (Peprotech, 315-20). After 5 h medium was removed, cells washed with 1× PBS, lysed and luciferase activity detected using the dual-luciferase assay and Glomax system (Promega). To compare Wnt5a secretory abilities by wild-type and *Rab8a*^−/−^ MEFs, cells were simultaneously transfected with pcDNA-WNT5A, Topflash and Renilla luciferase in Wnt-free lactalbumin hydrolysate (SAFC Biosciences, 58901-C) in DMEM for 16-18 h. Topflash activity was detected in cell lysates and normalized to intracellular Renilla luciferase. Data represent three independent experiments with comparable transfection efficiencies.

### Co-immunoprecipitation, western blot and GST pull-down

Immunoprecipitations were performed with anti-Flag M2 affinity gel (Sigma, A2220) using 2 mg total lysates extracted from 3×Flag-GPR177 stable HeLa cells. Reactions were incubated for 8 h at 4°C, washed three times with buffer containing 1% Triton X-100 and eluted with 40 μl 3×Flag peptides. Western blot and GST pull-down procedures have been described previously (Gao and Kaestner, 2010; Gao et al., 2009; Sakamori et al., 2012). The wash buffer comprised 50 mM Tris HCl (pH 7.5), 150 mM NaCl, 1 mM EDTA and 1% Triton X-100.

For GST pull-down, GST, GST-RAB8A, GST-CDC42 and GST-JFC-D1 fusion proteins were expressed in BL21 cells induced by 0.5 mM IPTG and cultured at room temperature for 18 h (Sakamori et al., 2012). The bacterial cells were then resuspended in 2 ml 1× PBS with 1% Triton X-100, 0.1 mg/ml lysozyme (Sigma, L6876), 1 mM PMSF and 1× bacterial protease inhibitors (Sigma, P8465), incubated on ice for 30 min followed by sonication. GST protein-containing lysates thus collected were incubated with pre-swollen glutathione-agarose beads (Molecular Probes, G-2878) at 4°C for 1 h. The beads were washed three times with 1× PBS. To check GST-protein expression, a portion of GST-protein-conjugated beads was denatured in 4× LDS (Life Technologies, NP0007) at 70°C for 15 min and subjected to SDS-PAGE followed by Coomassie Blue staining (Invitrogen, LC6060). Comparable amounts of beads were incubated with 1 mg cell lysates from 3×Flag-GPR177 stable HeLa cells for 1 h at 4°C. Beads were washed with PBS containing 1% Triton X-100 and mammalian protease inhibitors (Roche, 11 697 498 001), denatured in 4× LDS at 70°C for 15 min, and subjected to anti-Flag western blot analysis. Data represent three independent experiments.

### β-galactosidase staining, confocal immunofluorescence, immunohistochemistry, RNA *in situ* hybridization and quantitative RT-PCR

For β-galactosidase staining, tissue sections or whole-mount organoids in chamber slides were rinsed with 1× PBS, fixed in fixative (1% formaldehyde, 0.2% glutaraldehyde, 2 mM MgCl_2_, 5 mM EGTA and 0.02% NP40) for 15 min, washed in PBS three times at room temperature, and stained with staining solution comprising 5 mM K_3_Fe(CN)_6_, 5 mM K_4_Fe(CN)_6_, 2 mM MgCl_2_, 0.01% sodium deoxycholate, 0.02% NP40, 1 mg/ml X-Gal (Fisher Scientific, 50-213-181) overnight at 37°C. The samples were rinsed with PBS and mounted for imaging with a Nikon TE2000 microscope. Data represent three independent experiments.

Procedures for immunofluorescence, immunohistochemistry and *in situ* hybridization have been described previously (Gao and Kaestner, 2010; Gao et al., 2009; Sakamori et al., 2012). A complete list of antibodies is provided in the supplementary material Methods. *Wnt3a* and *Wnt3* RNA *in situ* probes were as described (Gregorieff et al., 2005; Roelink and Nusse, 1991). Quantitative RT-PCR is described in the supplementary material Methods and Table S1.

### Biotinylation assay

Cells were grown to ∼90% confluence in 10-cm dishes, washed three times with cold 1× PBS and incubated with 10 ml cold Biotin solution with gentle rocking at 4°C. Biotinylation was quenched after 30 min and cell lysates collected as per manufacturer's instructions (Pierce, 89881). Then, 300 μg cell lysates was added to 100 μl Neutravidin beads (Pierce) for 1 h at room temperature, washed four times with the wash buffer provided and eluted in 4× LDS containing 50 mM DTT at 70°C for 15 min. The supernatant was collected and immunoblot analysis performed using anti-Frizzled (1-10), anti-Lrp6, anti-phospho-Lrp6 (Ser1490), anti-N-cadherin and anti-Gpr177 (see supplementary material Methods). Data represent three independent experiments.

### Wnt5a/b secretion assay

MEFs were grown to ∼90% confluence in 10-cm dishes, washed three times with 1× PBS and cultured in 10 ml 1× lactalbumin hydrolysate (SAFC Biosciences, 58901-C) in DMEM. After 24 h, medium was collected in 50 ml Falcon tubes and centrifuged at 10,000 ***g*** for 10 min. Supernatant was loaded onto an Amicon Ultra-15 centrifugal filter system (Millipore, UFC 901024, 10K MWCO) and centrifuged at 5000 ***g*** at room temperature for 1 h. The concentrate (∼100 μl) was collected and subjected to immunoblot analysis using anti-Wnt5a/b and anti-histone H3 (see supplementary material Methods).

### Transmission electron microscopy (TEM) analysis and Gpr177 immunogold labeling

TEM procedures have been described previously (Gao et al., 2011; Sakamori et al., 2012). For Gpr177 immunogold labeling and TEM analysis, duodenal and jejunal tissues were dissected from wild-type (*n*=3) and *Rab8a^−/−^* (*n*=2) mice and immediately fixed as ∼1 mm fragments in 2.5% paraformaldehyde in cacodylate buffer (pH 7.4) overnight. The tissue was sliced to 100-200 μm thickness on a Vibratome and frozen between two brass ‘top hats’ in a HPM010 (Abra Fluid) at 5000 psi at –180°C. Next, the frozen tissue was transferred to frozen glass-distilled 100% acetone and dehydrated at –90°C for 48 h. The tissue was then infiltrated with HM-20 lowicryl (Electron Microscopy Sciences) and polymerized with 360 nm light at –50°C in a dry nitrogen environment. Tissue sections (60 nm thick) containing Paneth cells were immunolabeled with Gpr177 antibody (Fu et al., 2009) at 1:250 to 1:50 in 5% BSA, 0.1% cold water fish gelatin in PBS (pH 7.4). No-primary-antibody control and Gpr177-deficient cells were used in initial tests to optimize labeling conditions. Images shown were immunolabeled with 1:100 primary antibody. Stable antigen-antibody complexes were detected with protein A conjugated to either 15 nm gold colloids or 20 nm gold colloids (Electron Microscopy Sciences). Imaging was performed with an FEI Tecnai-12 microscope at 80 keV using a nominal magnification of 6500×. Montage images were collected using serial EM and stitched together with the IMOD subroutine Blendmont (Kremer et al., 1996; Mastronarde, 2005). Each image has a pixel dimension of ∼3 nm, such that each spherical gold particle should fill five pixels and resizing of the images should provide information that reveals the approximate volume the gold would occupy in the images. Immunogold particles were counted manually, excluding particles within the nuclear area. Area and perimeter of individual Paneth cells were measured using Photoshop CS (Adobe). Numbers of immunogold particles in each subcellular compartment per unit area (for ER and Golgi) or per unit length (for plasma membrane) were calculated and compared by *t*-test. Data were collected from three independent labeling experiments, performed twice on two pairs of independent wild-type and knockout tissues, and once on a wild-type mouse tissue.

### Quantifications and statistical analyses

Quantifications were performed using either two-tailed, unpaired or paired one-tailed Student's *t*-test on the basis of experimental setups. Mean values are shown, with error bars representing s.e.m. Western blots were quantified using ImageJ. β-gal staining areas and intensities were measured and quantified with the ‘color deconvolution’ plug-in provided in ImageJ. Non-linear regression analysis was used for curve fitting of the frequency of instantaneous speeds of GPR177-mCherry vesicles in MEFs. The histograms were fitted to a single Lorentzian distribution. All graphs were constructed using GraphPad Prism 5.

## Supplementary Material

Supplementary Material
