# Supplementary material for: Rab8a vesicles regulate Wnt ligand delivery and Paneth cell maturation at the intestinal stem cell niche
Source: Development. 2015 Jun 15;142(12):2147–62. doi: 10.1242/dev.121046 (PMC4483769; doi:10.1242/dev.121046)
Supplement: Supplementary Material [file supp_142_12_2147__index.html]

Rab8a vesicles regulate Wnt ligand delivery and Paneth cell maturation at the intestinal stem cell niche — Supplementary Material 

# Rab8a vesicles regulate Wnt ligand delivery and Paneth cell maturation at the intestinal stem cell niche

## DEV121046 Supplementary Material

- Supplementary Material
