# Supplementary material for: Rab8a vesicles regulate Wnt ligand delivery and Paneth cell maturation at the intestinal stem cell niche
Source: Development. 2015 Jun 15;142(12):2147–62. doi: 10.1242/dev.121046 (PMC4483769; doi:10.1242/dev.121046)
Supplement: Supplementary Material [file supp_dev.121046_DEV121046supp.pdf]

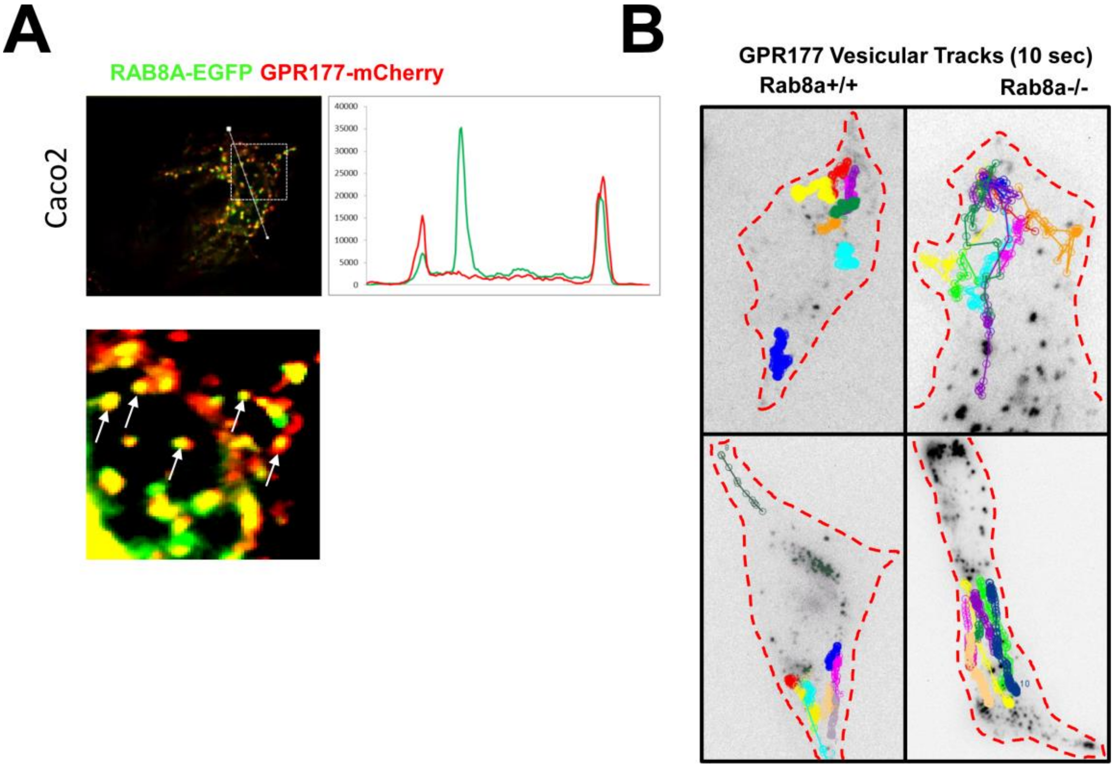

Fig. S1. GPR177 traffics through RAB8A vesicles, absence of which affects GPR177 trafficking dynamics. (A) Live cell imaging of GPR177-mCherry and EGFP-RAB8A in Caco2 cells. Line scan shows vesicles with both signals. **(B)** Individual vesicle tracks show vesicular movements within 10 sec. Tracks in different colors represent different tracks in designated cells with comparable morphology. The speed and directionality of vesicle movement in cells were analyzed (please see main text and Fig. 1F).

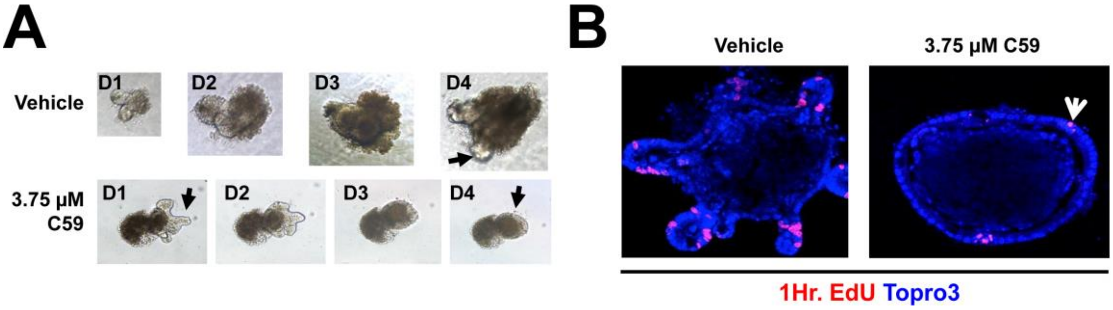

Fig. S2. Porcupine inhibitor C59 further abolished the growth of surviving *Rab8a*$^{-/-}$ organoids. (A) 3.75 μM C59 caused regression of epithelial buds in surviving *Rab8a*$^{-/-}$ organoids compared to vehicle treated ones. **(B)** C59 treatment inhibited proliferation of *Rab8a*$^{-/-}$ organoids.

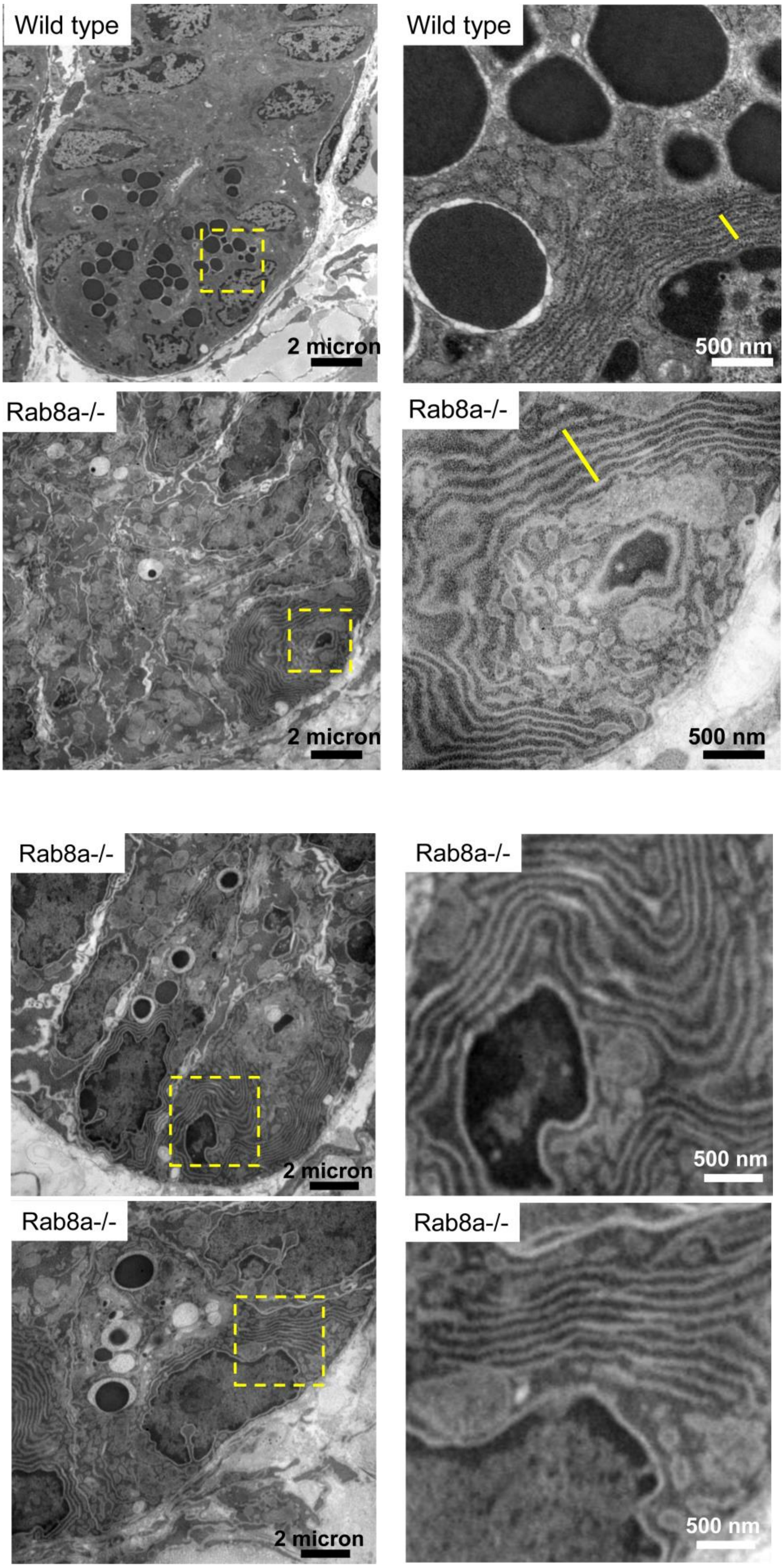
Wild type
2 micron
Wild type
500 nm
Rab8a-/-
2 micron
Rab8a-/-
500 nm
Rab8a-/-
2 micron
Rab8a-/-
500 nm
Rab8a-/-
2 micron
Rab8a-/-
500 nm

Figs S3 and S4. TEM micrographs show significant expansions of ER cisternae in *Rab8a*$^{-/-}$ Paneth cells. Straight yellow lines in Fig. S3 indicate the thicknesses of 5 ER cisternal stacks in wild type and *Rab8a*$^{-/-}$ Paneth cells. Fig. S4 shows additional *Rab8a*$^{-/-}$ Paneth cells, all of which had the similar ER morphology.

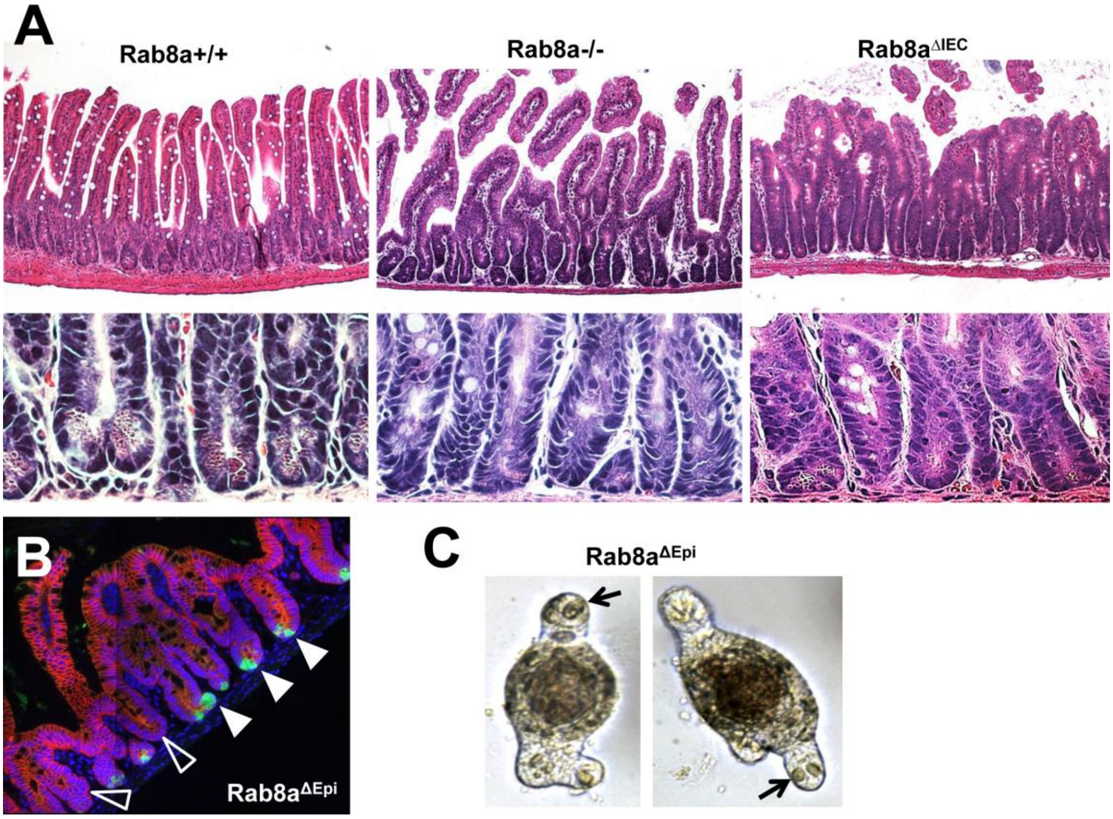

Fig. S5. *Rab8a$^{\Delta IEC}$* intestines showed less severe Paneth cell phenotype. (A) *Rab8a$^{\Delta IEC}$* crypts contained more mature Paneth cells than *Rab8a$^{-/-}$* crypts. **(B)** *Rab8a$^{\Delta IEC}$* intestines showed continuous stretches of Paneth cell-containing crypts (solid arrowheads), adjacent to Paneth cell-deficient crypts (empty arrowheads). **(C)** Some *Rab8a$^{\Delta IEC}$* organoids showed stronger organoid forming capacity than *Rab8a$^{-/-}$* organoids, as judged by larger buds containing Paneth cells (arrow).

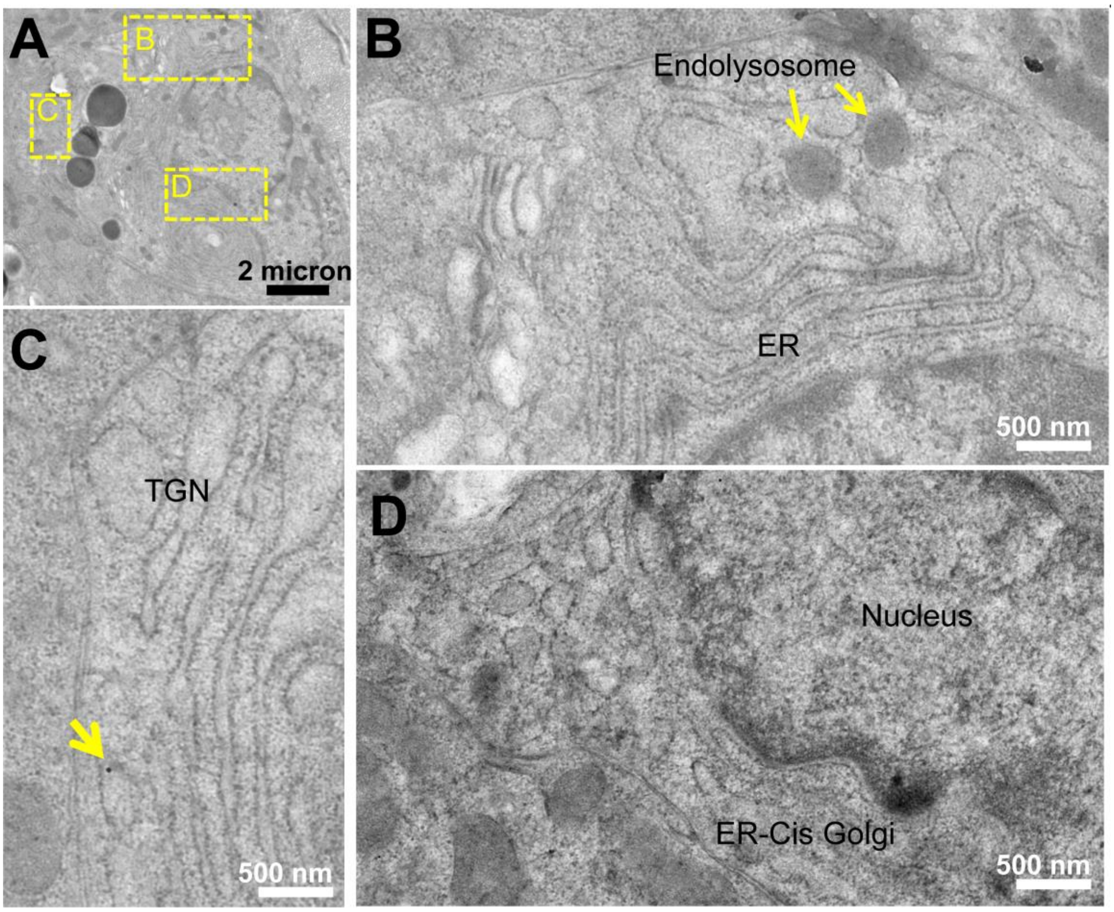

Fig. S6. TEM micrographs of Gpr177 immunogold labeling in Gpr177-deficient intestinal epithelial cells (*Gpr177$^{fl/-}$;Vil-Cre*). (A) Gpr177-deficient cells showed an overall lack of gold particles with immunogold labeling of Gpr177. **(B-E)** Areas containing ER, Golgi, endolysosomal compartments are shown in higher magnification. Arrow points to an occasionally detected gold particle. Quantification from 9 wild type and 7 Gpr177-deficient cells suggested 90%, 91%, and 93% reduction of gold particle numbers in ER, non-ER vesicular compartment, and plasma membrane, respectively.

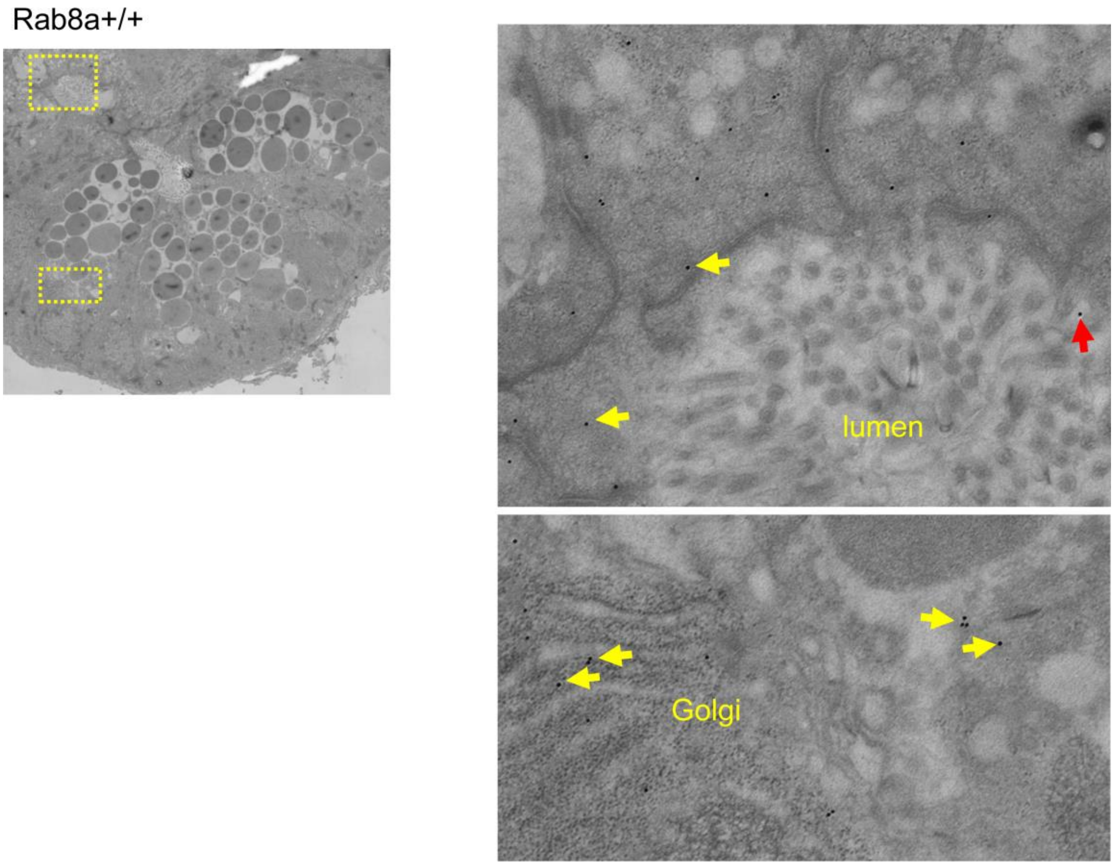

Fig. S7. TEM micrographs of Gpr177 immunogold labeling of *Rab8a*$^{+/+}$ crypts. In wild type cells, abundant gold particles were detected in Golgi and apical peripheral regions (arrows). Red arrow points to a gold particle at apical surface.

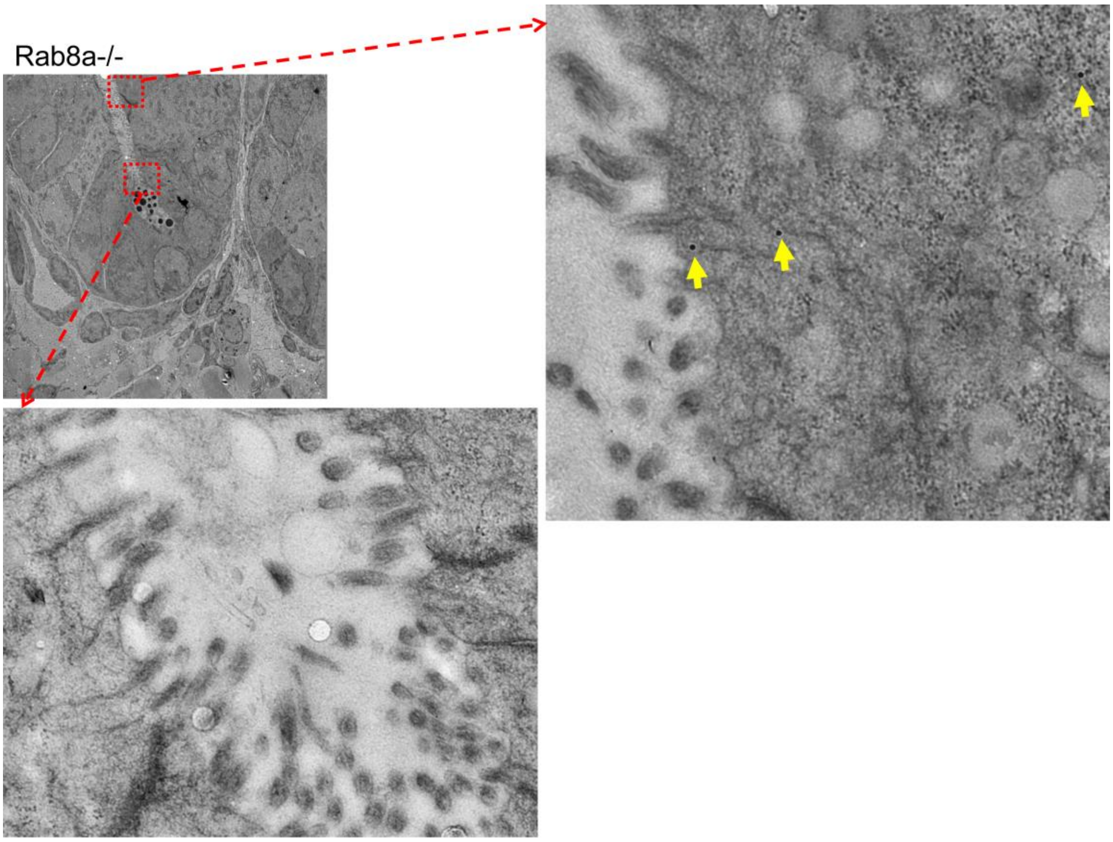

Fig. S8. TEM micrographs of Gpr177 immunogold labeling of *Rab8a*$^{-/-}$ crypts. A single Paneth cell remained in this Rab8a$^{-/-}$ crypt. Only occasional gold particles (arrows) were observed in apical domains.

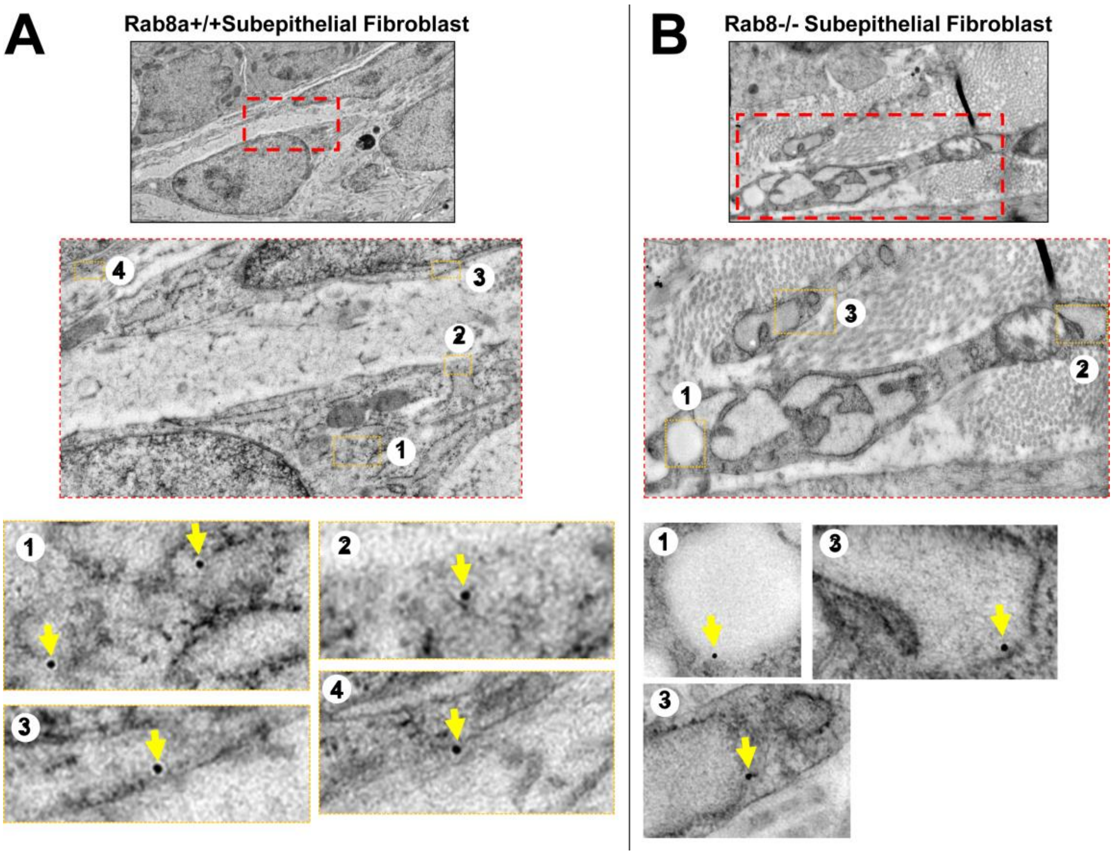

Fig. S9. Reduced Gpr177 localization adjacent to plasma membrane of Rab8a$^{-/-}$ submucosal stromal cells.

(A) Wild type intestinal subepithelial fibroblasts show Gpr177 gold particles in Golgi-vesicles (1), cell peripheral (2), and at plasma membranes (3)(4). **(B)** Rab8a$^{-/-}$ subepithelial fibroblasts show reduced particles at peripheral regions near plasma membranes. The increased vacuolar structures, similar to previously reported lysosomes, frequently contained gold particles.

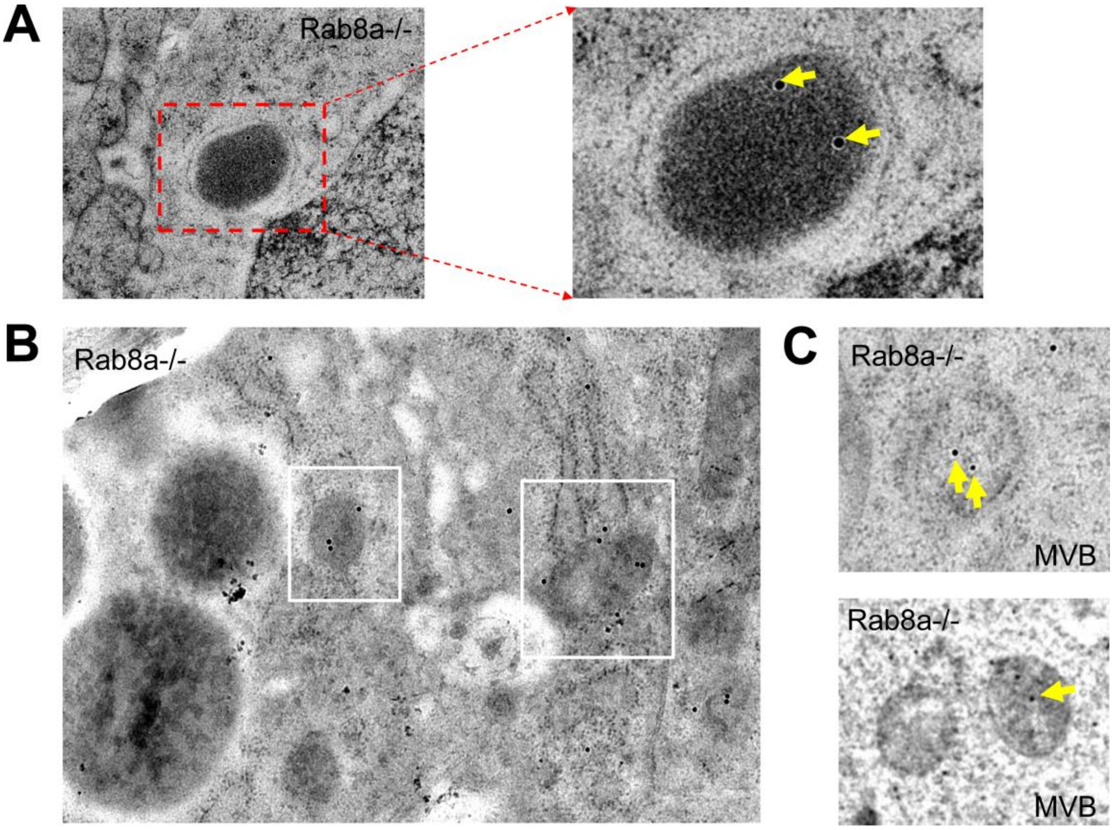

Fig. S10. Localization of Gpr177 to lysosome and MVBs in Rab8a$^{-/-}$ crypt cells. (A-C) Gpr177 gold particles were frequently detected in lysosomes (A and B), and MVBs (C). Wild type crypt cells contain few gold particles in lysosome and MVBs (please see main text and Fig. 6C-D).

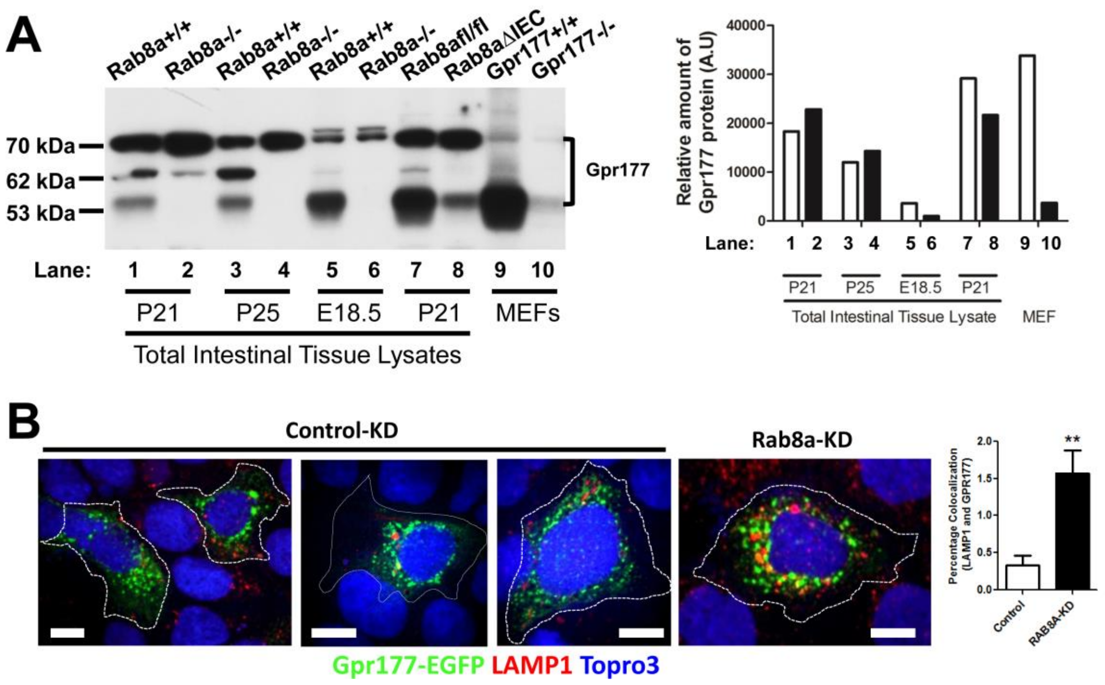

Fig. S11. Increased Gpr177 localization to endolysosomes in Rab8a deficient cells. (A) Western blots for Gpr177 were performed on total intestinal or MEF cell lysates. Genotypes and ages are indicated for each lane. Predicted molecular weight for full-length mouse Gpr177 is 62 kDa. Note the residual protein at 53 kDa for Gpr177$^{-/-}$ MEF lysate (lane 10) reflect incomplete deletion by retrovial Cre in culture. Data represent 3 independent experiments. **(B)** 2-fold increase of GPR177 (green) localization in the lysosome (red) was observed in RAB8A-KD Caco2 cells. N=20 cells were analyzed for colocalization assay.

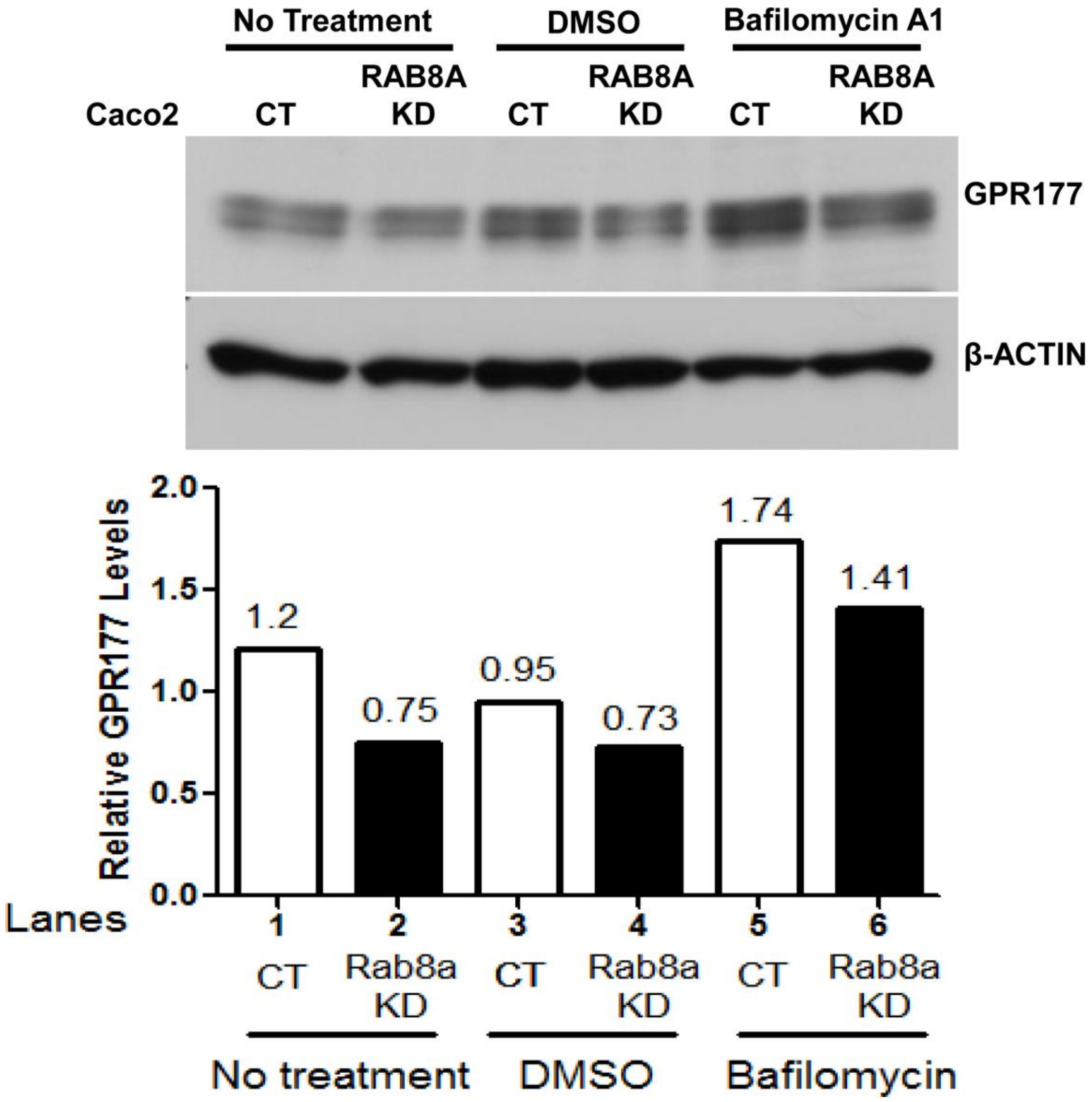

Fig. S12. Bafilomycin A1 treatment increased Gpr177 level. Control and *RAB8A*-KD Caco2 cells treated with Bafilomycin A1, a lysosome inhibitor, increased total GPR177 levels.

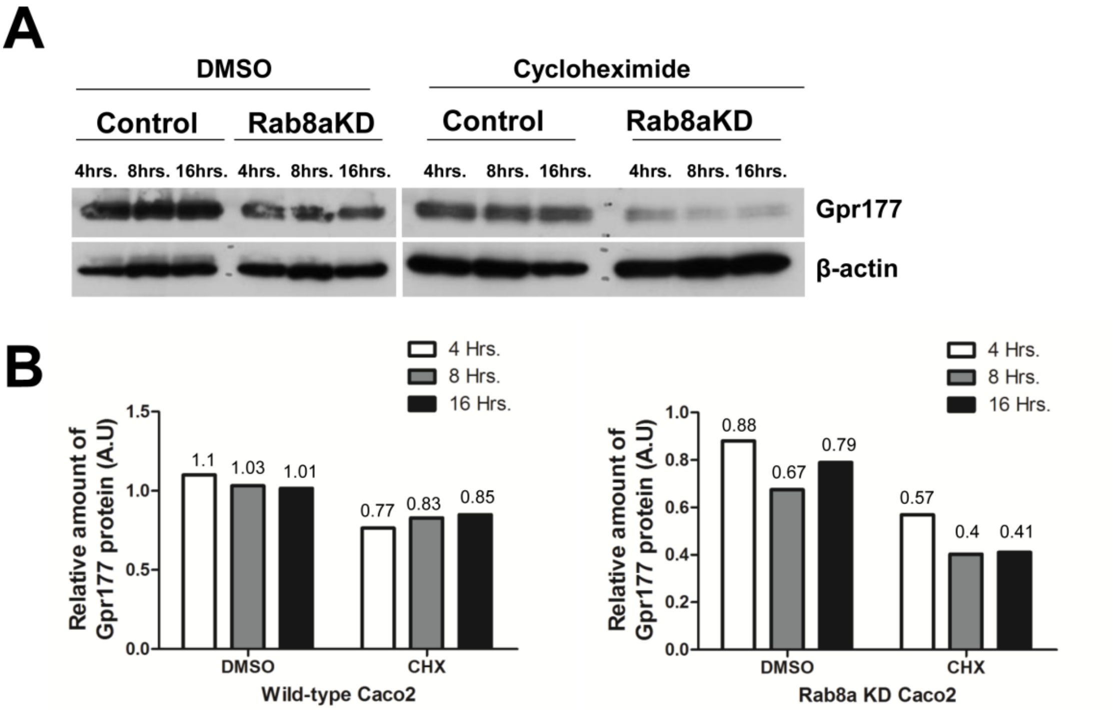

Fig. S13. Inhibition of protein synthesis by cycloheximide treatment showed faster degradation of Gpr177 in *RAB8A-KD* Caco2 cells.

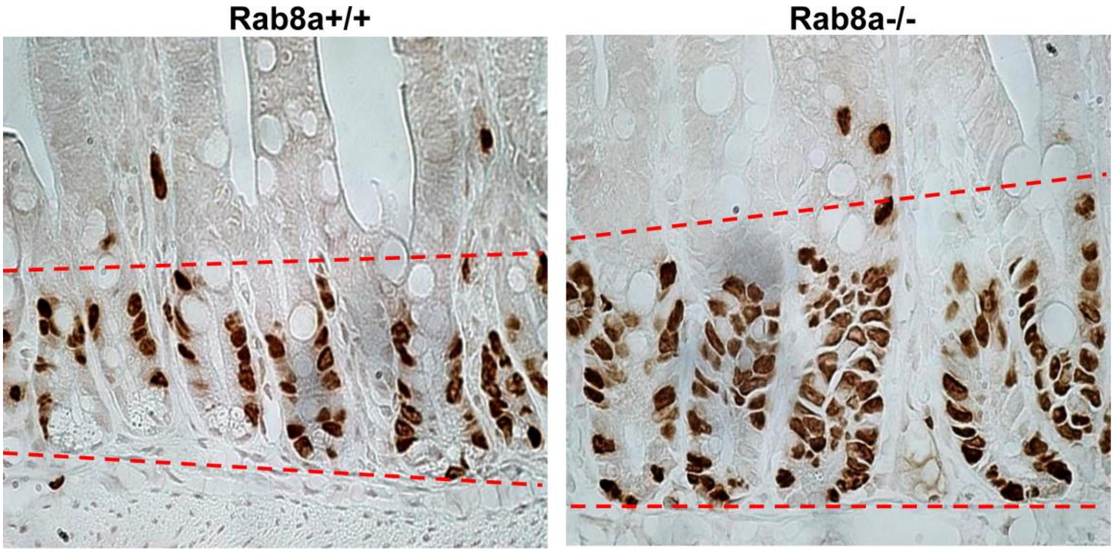

Fig. S14. Immunohistochemistry for BrdU (1 hr.) detected an expanded transit amplifying epithelial cell compartment in Rab8a$^{-/-}$ intestine. Dotted lines mark the proliferative regions of small intestinal epithelia in wild type and knockout littermate mice.

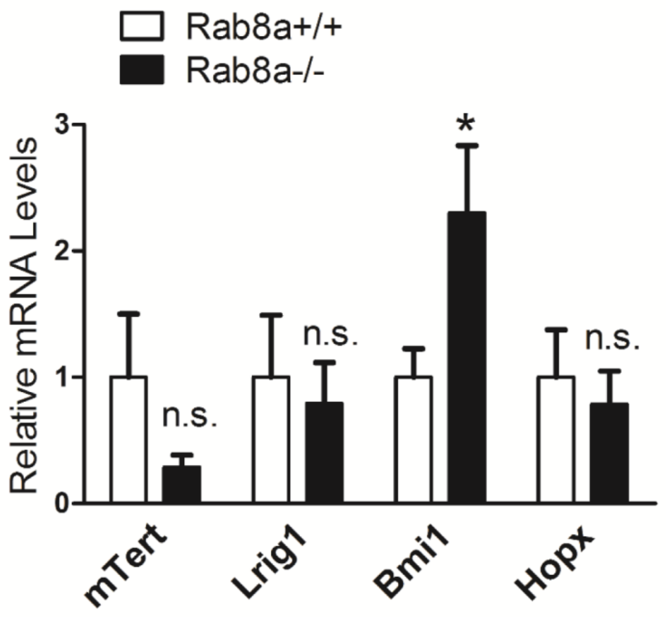

Fig. S15. Quantitative RT-PCR identified increased *Bmi1* gene expression in Rab8a$^{-/-}$ intestines.

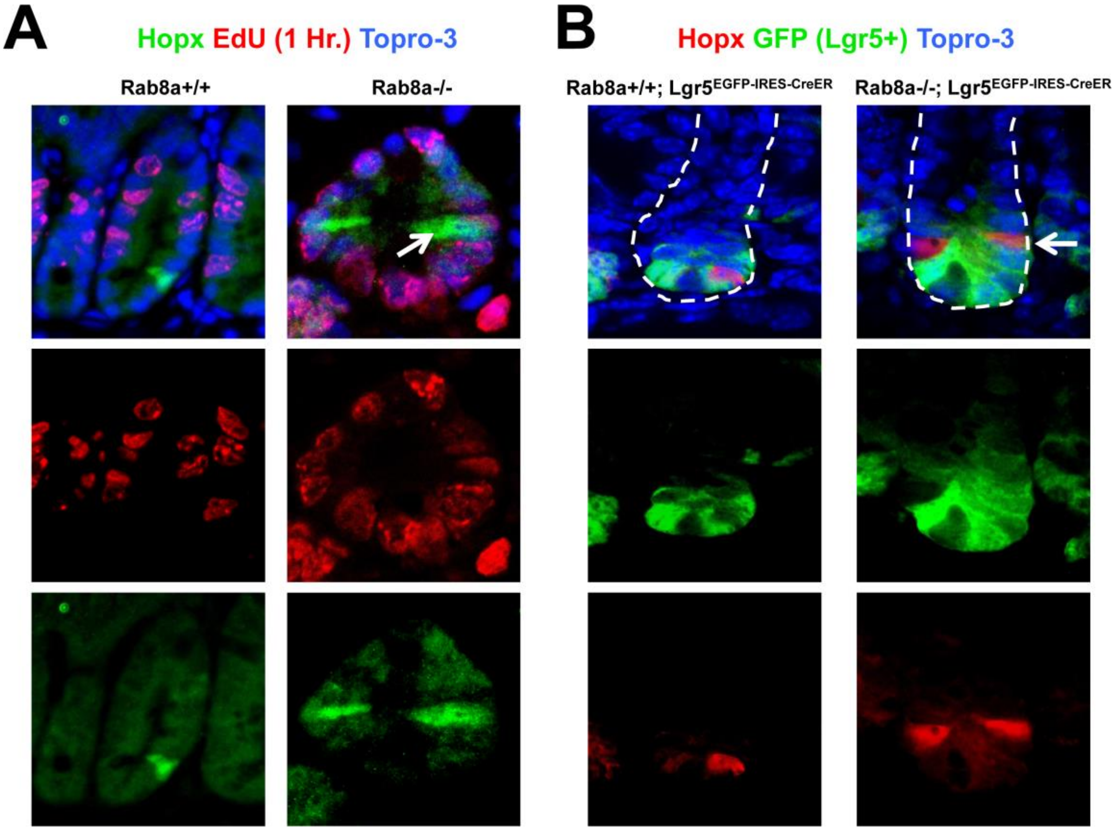

Fig. S16. Increased Hopx+ cells in Rab8a$^{-/-}$ intestines. (A) Hopx (green) and EdU (1 hr., red) co-staining detected proliferative Hopx^{+} cells in Rab8a$^{-/-}$ crypts but rarely in wild types. **(B)** Lgr5 (EGFP, green) and Hopx (red) co-staining showed increased numbers of both cell types in Rab8a$^{-/-}$ crypts.

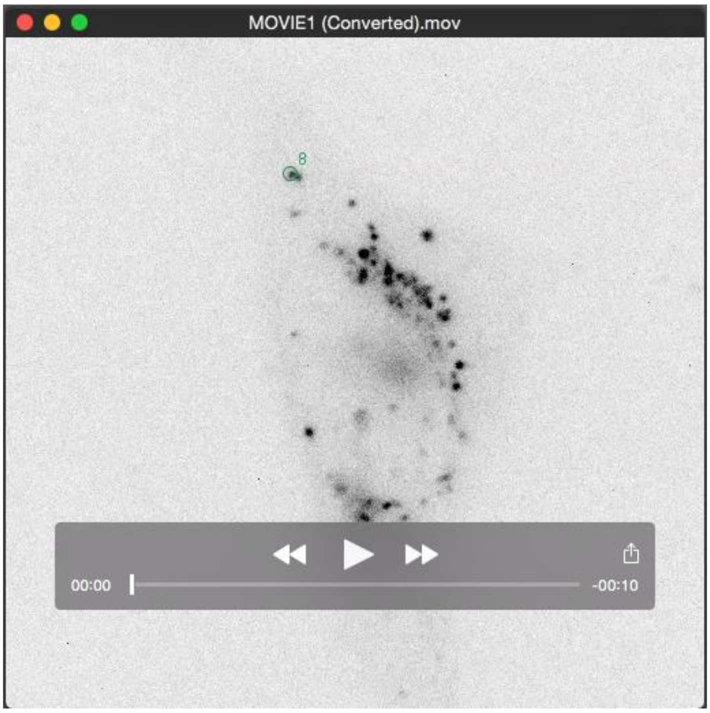

Movie 1. A representative track of a single Gpr177-mCherry vesicle movement in a wild type MEF cell. The instantaneous speeds were computed for each step of the tracks in wild type cells (total 1679 steps). Note the vesicle was eventually delivered to plasma membrane.

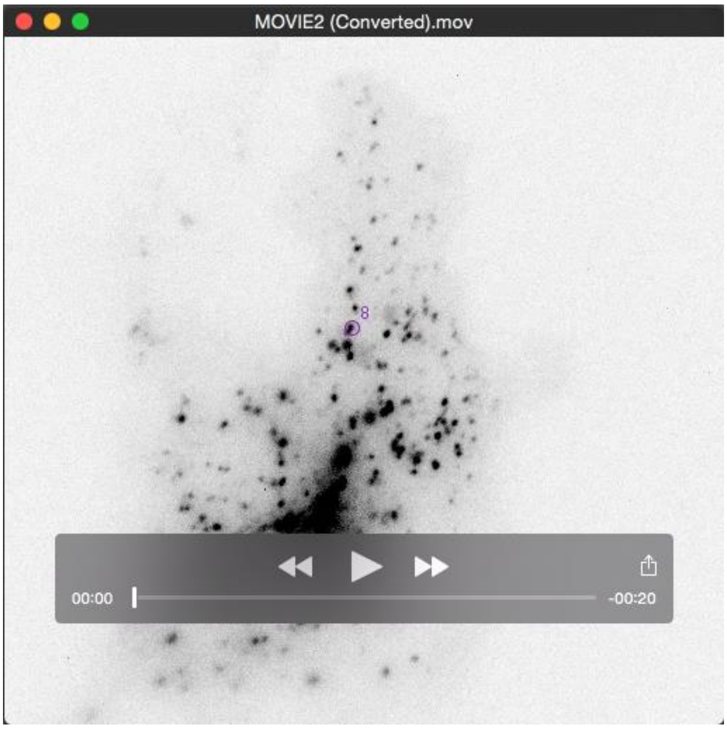

Movie 2. A representative track of a single Gpr177-mCherry vesicle movement in a Rab8a$^{-/-}$ MEF cell. The instantaneous speeds were computed for each step of the tracks in Rab8a$^{-/-}$ cells (total 995 steps).

Supplementary Materials and Methods

Antibodies. Antibodies used for immunofluorescence staining are as follows. Wnt5a (Cell Signaling, 2530), Frizzled (1-10) (Santa cruz, 9169), Lrp6 (Cell Signaling, 3395), p-Lrp6 (Cell Signaling, 2568), N-Cadherin (BD Transduction 610920), Lysozyme (Biogenex, AR024-5R), Hopx (Santa-Cruz, sc-30216), GFP (Invitrogen, A11122 and Abcam, ab6673), E-Cadherin (BD Transduction Laboratories, 610182), Histone H3 (Cell Signaling, 12648), Phospho-Histone H3 (Millipore, 06-570), 5-Bromodeoxyuridine (Abdserotec, OBT0030), β-catenin (Abcam, ab16051), β-actin (Santa Cruz, sc-47778), C-myc (Millipore, CBL 430), , Rab8 (BD Transduction Laboratories, 610845), Sox9 (Millipore, AB5535), Mmp7 (Santa Cruz, 8832), Flag (Sigma, F1804), Tcf-1 (Cell Signaling, 2203S), Tcf-4 (Cell Signaling, 2565S), Gpr177 (Fu et al., 2009), Rab5 (abcam, ab13253), Rab7 (Cell Signalling, 9367P), Rab9 (Cell Signalling, 5118), Rab11 (BD Tansduction, 610656), Vps35 (abcam, ab10099), LAMP1 (abcam, ab25630),. Nuclei were stained with Topro-3 (Invitrogen, T3605), and 5-ethynyl-2'-deoxyuridine (EdU) staining was performed using Click-iT EdU Alexa Fluor 555 Imaging Kit (Invitrogen, C10338) using manufacturer's protocol.

Cell culture, plasmids, transfection, and lentiviral shRNA knockdown. HeLa and Caco2 cells were obtained from ATCC and maintained according to manuals. Wild-type and RAB8AKD Caco2 cells were treated with 0.1 μM of Bafilomycin A1 (Sigma, B1793) for 18 hours and 2μg/ml of Cyclohexamide (Sigma, C7698) for indicated time. Renilla luciferase (pRL-CMV), Wnt3a-Guassia luciferase, pEGFP-RAB8A, and pGEX-KG-RAB8A have been described previously (Chen et al., 2009; Gao et al., 2003; Knodler et al., 2010a; Knodler et al., 2010b; Sakamori et al., 2012b). truncated (ΔC44) GPR177 coding sequences were tagged with 3×Flag by inserting the CDS into pQCXIP retroviral vector pre-engineered to carry N-terminal 3×Flag. GPR177-mCherry was constructed by inserting the GPR177 cDNA into pmCherry-N1 vector to carry mcherry tag at the C-terminus. Topflash plasmid (12456), pcDNA-WNT5A (35911) pcDNA3-ShhN-Ren (37677) and pGEX4T-1-CDC42 (12175) were obtained from Addgene. SEcreted Alkaline Phosphatase (SEAP) and Metridia luciferase constructs were provided with the Great EscAPe Fluorescence detection kit (Clontech, 631704). Transient transfections were performed with Lipofectamine 2000 (Life Technologies) using manufacturer's recommendations for plasmid

concentrations except Renilla-luciferase which was used at a concentration of 5-10 ng per 500μl of transfection system. Stable cell lines expressing Flag- or mCherry-tagged Gpr177 in pQCXIP vector were established using lentiviral transduction under puromycin selection pressure. The Stable RAB8A knockdown Caco2 cells were established using lentiviral shRNA transduction particles (Sigma, TRCN0000048213) and puromycin selection following procedures as described earlier (Gao and Kaestner, 2010). Live cell images were acquired by a Zeiss LSM 510 confocal or a Zeiss Cell Observer Spinning Disk confocal microscope. pGEX4T-1, pGEX4T-1-RAB8A and pGEX4T-1-JFC1D1 plasmids are described elsewhere (Feng et al., 2012; Sakamori et al., 2012a) pQCXIH-Cre was made by inserting cDNA of Cre into pQCXIH (Clontech, 631516). Cell experiments were performed in triplicates, repeated multiple times, and only consistent results were presented.

Derivation of wild type, Rab8a$^{-/-}$, Rab8b-Knockdown, and Gpr177$^{-/-}$ MEFs. The trunk of E12.5-13.5 wild type, Rab8a$^{-/-}$, or Gpr177 $^{fl/fl}$ embryos were diced into small pieces with fine aseptic scissors and treated with Trypsin-EDTA at 37°C for 20 mins. The tissue remnants were pipetted up and down, centrifuged at 1,000 rpm for 3 mins. Pellets were plated in 1× DMEM (Dulbecco's Minimal Essential media) with 10% FBS, 5% Penicillin/Streptomycin and 10 μg/mL Gentamycin. To knockdown Rab8b, wild type or Rab8a$^{-/-}$ MEFs were infected with lentiviral shRab8b particles (sequences below) and selected by Puromycin (3μg/ml) for 2 days. To derive Gpr177$^{-/-}$ MEFs, Gpr177$^{fl/fl}$ MEFs were infected with retroviral CMV-Cre and selected by Hygromycin B (500 μg/ml) for 3 weeks. For Cre retroviral production, pQCXIH-Cre and pVSV-G (Clontech, 631457) were co-transfected into GP2-293 cells (Clontech 631458). After 48 hrs, cell culture medium was harvested and ultracentrifuged at 30,000 g for 2 hrs to concentrate retroviral Cre particles.

ShRNA sequences for Rab8b knockdown.

shRab8b 1	CCGGGCCAAGAACTAACAGAACTTTCCATGGAAAGTTCTGTTAGTTCTTGGCTTTTTG
shRab8b 2	AATTCAAAAAGCCAAGAACTAACAGAACTTTCCATGGAAAGTTCTGTTAGTTCTTGGC

Intestinal organoid culture. Modified procedures, adapted from orginal report (Sato et al., 2011), have been described elsewhere (Perekatt et al., 2014; Sakamori et al., 2014; Yu et al., 2014). Proximal one third of the intestines were dissected and flushed

with 1× Phosphate Buffer Saline (PBS), cut open and fragmented into smaller pieces (~2 cm sizes). Tissue fragments were then rinsed 3 times in cold 1× PBS followed by 2 washes in cold chelating buffer (2 mM EDTA in PBS) at 4°C for 5 minutes and 40 minutes respectively. Intestinal fragments were then vigorously re-suspended in cold chelation buffer and allowed to flow through 70 μm filter (BD Falcon, 352350) into pre-cooled 1× PBS. A pellet of crypts was obtained by centrifuging this flow-through suspension at 200 g for 3mins at 4°C. The pellet was then washed twice and resuspended in cold 1× PBS for counting. 100~200 crypts suspended in matrigel (BD Biosciences, 356231) were plated into each well of pre-warmed (at 37 °C) 24-well plates. After allowing the matrigel to solidify at 37°C for 10 minutes, 500 μl of ENR organoid culture medium was added to each well (Sato et al., 2011). Working ENR medium contains 2× N2 supplement (Life Tech Gibco. 17502-048), 0.5× B27 (Life Tech Gibco. 17504-044), 1 mM N-Acetyl Cysteine (Sigma, A9165), 0.05 μg/ml of EGF (Life Technologies, PMG8043), 0.1 μg/ml of Noggin (Peprotech, 250-38) and 1μg/ml of R-Spondin (R & D Systems, 3474-RS-050)] made in Basal Culture medium [Advanced DMEM/F12 (Life Technologies, 12634-010) with 1× Penicillin/Streptomycin (Life Technologies, 15140-122), 1× Glutamax (Life Technologies, 35050-061) and 10mM HEPES Buffer (Life Technologies, 15630-080)]. To rescue Rab8a$^{-/-}$ organoids, culture medium with 100 ng/mL mouse recombinant Wnt3a (R & D Systems, 1324-WN-002), 3μM of CHIR99021 (Stemgent, 04-0004) or C-59 (Cellagen, C7641-2) was added daily to the culture. Organoid survival results and images of β-galactosidase staining of organoids are from 3 independent experiments. Wnt3a and CHIR99021 rescue experiments were repeated twice.

Quantitative RT-PCR. Quantitative RT-PCR has been described earlier (Gao and Kaestner, 2010; Gao et al., 2009; Sakamori et al., 2012b; Sakamori et al., 2014), with a list of primers provided in Table S1. Threshold cycle (C_t) values obtained for each gene were normalized to C_t values obtained for either *β-actin* or *Hypoxanthine-guanine phosphoribosyl transferase*. Data was obtained from 3 independent biological samples with 3 technical replicates.

Table S1. Quantitative RT-PCR primers

Gene	Forward	Reverse
c-Myc	GTGCTGCATGAGGAGACACC	CAGGGGTTTGCCTCTTCTCC
Tcf4	AGCCCGTCCAGGAACTATG	TGGAATTGACAAAAGGTGGA
Tcf1	AGCCTCAACCCCCGCTGCAT	CTTGCTTCTGGCTGATGTCC
Axin2	TGAGATCCACGGAAACAGC	GTGGCTGGTGCAAAGACAT
Gpr177	CAAATCGTTGCCTTTCTGGT	CGCCAGCCATCTTGTTTTAT
Lyz	GGTGGTGAGAGATCCCCAAG	CAGACTCCGCAGTTCCGAAT
Mmp7	CTTACAAAGGACGACATTGCAG	AGTGCAGACCGTTTCTGTGAT
Defa5	TATCTCCTTTGGAGGCCAAG	TTTCTGCAGGTCCCAAAAAC
Wnt3	CTTCTAATGGAGCCCCACCT	GAGGCCAGAGATGTGTACTGC
Wnt3a	GGAATGGTCTCTCGGGAGTT	CTTGAGGTGCATGTGACTGG
Wnt2b	CCGTGTAGACACGTCCTGGT	TGATGTCTGGGTAGCGTTGA
Wnt5a	GACAGGCATCAAGGAATGC	GTCTCTCGGCTGCCTATTTG
Wnt9a	GGCGCTCTAGCAAGGATTT	CCAGACACACCATGGCATT
Wnt6	CGTGGAGATATCCGTGCAT	CCCATGGCACTTACACTCG
Ascl2	TCCAGTTGGTTAGGGGGCTA	GCATAGGCCCAGGTTTCTTG
Tert	AGCGGGATGGGTTGCTTTTAC	CACCCATACTCAGGAACGCC (Munoz et al., 2012)
Bmi1	GAGCAGATTGGATCGGAAAG	GCATCACAGTCATTGCTGCT (Sakamori et al., 2012a)
Lrig1	AAGGGAACTCAACTTGGCGAG	ACGTGAGGCCTTCAATCAGC (Munoz et al., 2012)
Hopx	CATCCTTAGTCAGACGCGCA	AGGCAAGCCTTCTGACCGC (Munoz et al., 2012)
Olfm4	GCCACTTTCCAATTTCAC	GAGCCTCTTCTCATACAC
β-actin	TTGCTGACAGGATGCAGAAG	CCACCGATCCACACAGAGTA
Hprt	AAGCTTGCTGGTGAAAAGGA	TTGCGCTCATCTTAGGCTTT

Supplementary references

Chen, B., Dodge, M. E., Tang, W., Lu, J., Ma, Z., Fan, C. W., Wei, S., Hao, W., Kilgore, J., Williams, N. S. et al. (2009). Small molecule-mediated disruption of Wnt-dependent signaling in tissue regeneration and cancer. *Nat Chem Biol* **5**, 100-7.

Feng, S., Knodler, A., Ren, J., Zhang, J., Zhang, X., Hong, Y., Huang, S., Peranen, J. and Guo, W. (2012). A Rab8 guanine nucleotide exchange factor-effector interaction network regulates primary ciliogenesis. *The Journal of biological chemistry* **287**, 15602-9.

Fu, J., Jiang, M., Mirando, A. J., Yu, H. M. and Hsu, W. (2009). Reciprocal regulation of Wnt and Gpr177/mouse Wntless is required for embryonic axis formation. *Proceedings of the National Academy of Sciences of the United States of America* **106**, 18598-603.

Gao, N., Davuluri, G., Gong, W., Seiler, C., Lorent, K., Furth, E. E., Kaestner, K. H. and Pack, M. (2011). The nuclear pore complex protein Elys is required for genome stability in mouse intestinal epithelial progenitor cells. *Gastroenterology* **140**, 1547-55 e10.

Gao, N. and Kaestner, K. H. (2010). Cdx2 regulates endo-lysosomal function and epithelial cell polarity. *Genes Dev* **24**, 1295-305.

Gao, N., White, P. and Kaestner, K. H. (2009). Establishment of intestinal identity and epithelial-mesenchymal signaling by Cdx2. *Dev Cell* **16**, 588-99.

Gao, N., Zhang, J., Rao, M. A., Case, T. C., Mirosevich, J., Wang, Y., Jin, R., Gupta, A., Rennie, P. S. and Matusik, R. J. (2003). The role of hepatocyte nuclear factor-3 alpha (Forkhead Box A1) and androgen receptor in transcriptional regulation of prostatic genes. *Mol Endocrinol* **17**, 1484-507.

Gregorieff, A., Pinto, D., Begthel, H., Destree, O., Kielman, M. and Clevers, H. (2005). Expression pattern of Wnt signaling components in the adult intestine. *Gastroenterology* **129**, 626-38.

Knodler, A., Feng, S., Zhang, J., Zhang, X., Das, A., Peranen, J. and Guo, W. (2010a). Coordination of Rab8 and Rab11 in primary ciliogenesis. *Proceedings of the National Academy of Sciences of the United States of America* **107**, 6346-51.

Knodler, A., Feng, S., Zhang, J., Zhang, X., Das, A., Peranen, J. and Guo, W. (2010b). Coordination of Rab8 and Rab11 in primary ciliogenesis. *Proceedings of the National Academy of Sciences of the United States of America* **107**, 6346-51.

Kremer, J. R., Mastronarde, D. N. and McIntosh, J. R. (1996). Computer visualization of three-dimensional image data using IMOD. *J Struct Biol* **116**, 71-6.

Mastronarde, D. N. (2005). Automated electron microscope tomography using robust prediction of specimen movements. *J Struct Biol* **152**, 36-51.

Meijering, E., Dzyubachyk, O. and Smal, I. (2012). Methods for cell and particle tracking. *Methods Enzymol* **504**, 183-200.

Munoz, J., Stange, D. E., Schepers, A. G., van de Wetering, M., Koo, B. K., Itzkovitz, S., Volckmann, R., Kung, K. S., Koster, J., Radulescu, S. et al. (2012). The Lgr5 intestinal stem cell signature: robust expression of proposed quiescent '+4' cell markers. *The EMBO journal* **31**, 3079-91.

Perekatt, A. O., Valdez, M. J., Davila, M., Hoffman, A., Bonder, E. M., Gao, N. and Verzi, M. P. (2014). YY1 is indispensable for Lgr5+ intestinal stem cell renewal. *Proc Natl Acad Sci U S A* **111**, 7695-700.

Roelink, H. and Nusse, R. (1991). Expression of two members of the Wnt family during mouse development--restricted temporal and spatial patterns in the developing neural tube. *Genes Dev* **5**, 381-8.

Sakamori, R., Das, S., Yu, S., Feng, S., Stypulkowski, E., Guan, Y., Douard, V., Tang, W., Ferraris, R. P., Harada, A. et al. (2012a). Cdc42 and Rab8a are critical for intestinal stem cell division, survival, and differentiation in mice. *The Journal of clinical investigation* **122**, 1052-65.

Sakamori, R., Das, S., Yu, S., Feng, S., Stypulkowski, E., Guan, Y., Douard, V., Tang, W., Ferraris, R. P., Harada, A. et al. (2012b). Cdc42 and Rab8a are critical for intestinal stem cell division, survival, and differentiation in mice. *J Clin Invest* **122**, 1052-65.

Sakamori, R., Yu, S., Zhang, X., Hoffman, A., Sun, J., Das, S., Vedula, P., Li, G., Fu, J., Walker, F. et al. (2014). CDC42 Inhibition Suppresses Progression of Incipient Intestinal Tumors. *Cancer Res* **74**, 5480-92.

Sato, T., van Es, J. H., Snippert, H. J., Stange, D. E., Vries, R. G., van den Born, M., Barker, N., Shroyer, N. F., van de Wetering, M. and Clevers, H. (2011). Paneth cells constitute the niche for Lgr5 stem cells in intestinal crypts. *Nature* **469**, 415-8.

Yu, S., Nie, Y., Knowles, B., Sakamori, R., Stypulkowski, E., Patel, C., Das, S., Douard, V., Ferraris, R. P., Bonder, E. M. et al. (2014). TLR sorting by Rab11 endosomes maintains intestinal epithelial-microbial homeostasis. *Embo J* **33**, 1882-95.
